# Dynamic shifts in trophoblast nucleos(t)ide metabolism, transport, and adenosine signaling during gestation and preterm birth

**DOI:** 10.1038/s41598-025-16183-2

**Published:** 2025-08-18

**Authors:** Mohammed Ali, Mariia Adler, Antonin Libra, Ivan Vokral, Rona Karahoda, Eva Cifkova, Miroslav Lisa, Jakub Tomek, Magdalena Novotna, Frantisek Staud, Lukas Cerveny

**Affiliations:** 1 Department of Pharmacology and Toxicology, Faculty of Pharmacy in Hradec Kralove, Hradec Kralove, Charles University, Hradec Kralove, Czech Republic; 2 Department of Chemistry, Faculty of Science, University of Hradec Kralove, Hradec Kralove, Czech Republic; 3 Institute of Clinical Biochemistry and Diagnostics, University Hospital Hradec Kralove, Hradec Kralove, Czech Republic

**Keywords:** Nucleotide and nucleoside metabolism in placenta, Adenosine receptors, Preterm birth, Nucleoside transporters, Placental gene expression, Cytotrophoblast and syncytiotrophoblast, Differentiation, Intrauterine growth, Molecular biology

## Abstract

Nucleos(t)ides are essential for DNA/RNA synthesis, energy metabolism, and signaling, yet their roles in placental development remain poorly understood. The placenta undergoes dynamic metabolic adaptations throughout gestation to support fetal growth. This study investigates gene expression shifts in nucleos(t)ide metabolism, transport, and adenosine signaling during placental development and in the pathological condition of spontaneous preterm birth (PTB). We analyzed gene expression in first-trimester (*n* = 10) and term (*n* = 10), and PTB (*n* = 10) human placentas, and in cytotrophoblast and syncytiotrophoblast stage in primary human trophoblasts (*n* = 3) and BeWo (*n* = 5) cells. For developmental context, rat placentas were examined at gestation days (GD) GD12, GD15, and GD20 (*n* = 5 per group) that correspond to early second trimester in the human placenta. We found that genes involved in nucleos(t)ide metabolism and adenosine signaling were dominantly upregulated from early gestation to term in the human placenta. PTB placentas revealed further elevation compared to the term placenta. Differentiation from cytotrophoblast to syncytiotrophoblast was accompanied by only minor changes. Pearson’s correlation analysis revealed strong gene-metabolite and gene-gene associations, highlighting an integrated metabolic network regulating placental function. Gene expression also differed among the tested GDs in the rat placenta. These findings demonstrate dynamic changes of nucleos(t)ide metabolism during healthy placental development and enhanced expression in PTB placentas, suggesting increasing needs for nucleos(t)ides during placental growth and metabolic shifts in the PTB placenta. Our data also indicate that nucleos(t)ide metabolism is preserved in both proliferative and differentiated states.

**Supplementary Information:**

The online version contains supplementary material available at 10.1038/s41598-025-16183-2.

## Introduction

The placenta is a metabolically active organ that facilitates nutrient and oxygen exchange between the mother and fetus, regulates immune tolerance, and supports fetal growth. These functions require high rates of cell proliferation, differentiation, and metabolic adaptation, particularly within the trophoblast lineage, which consists of stem-like cytotrophoblast cells (CTB) and a layer of differentiated syncytiotrophoblast (STB)^1–3^. Among the key metabolic pathways supporting these processes is nucleos(t)ide metabolism, which is critical for DNA and RNA synthesis, energy homeostasis, and cell signaling^4^. Although well characterized in highly proliferative tissues such as cancer^5^, its role in placental development remains poorly understood.

The placenta undergoes dynamic metabolic shifts throughout gestation, ensuring an adequate nucleotide supply for rapid trophoblast expansion, differentiation, and fetal-maternal communication^3^. Nucleos(t)ides can be synthesized *de novo*, a process requiring substantial ATP investment, or salvaged from pre-existing nucleotide pools, a more energy efficient alternative^6^ (both pathways are detailed in Supplementary Fig. 1 and Fig. 2). Both pathways are tightly regulated to balance cellular proliferation, differentiation, and adaptation to external stressors^7–10^. Adenosine, a nucleoside with unique signaling properties, interacts with adenosine receptors to regulate blood flow, immune responses, and trophoblast function^11–13^. While nucleos(t)ide metabolism and adenosine signaling have been extensively studied in oncogenesis^4,14^, their roles in placental development and pregnancy complications remain underexplored.

Altered placental metabolism is a hallmark of placental functions and is closely linked to pregnancy complications, including preterm birth (PTB), which remains a leading cause of neonatal morbidity and mortality^15,16^. Emerging evidence suggests that dysregulation of nucleos(t)ide metabolism may contribute to adaptive responses under stress conditions^4,17^.

Studies have reported elevated plasma adenosine levels by the end of pregnancy^18^, significant accumulation of adenosine and its metabolites in the placenta^19^, along with the expression of adenosine receptors and nucleoside transporters in the trophoblast^2,20^. These findings suggest a critical role for adenosine in placental function. Recent findings suggest that dietary adenosine supplementation enhances placental angiogenesis in piglets experiencing intrauterine growth restriction^7^ and nucleos(t)ide supplementation promotes immune responses and growth in infants with intrauterine growth restriction^21,22^. Notably, dysregulated levels of nucleosides and their metabolites in PTB placentas^23^ further suggest that nucleosides contribute to placental adaptation under pathological conditions. However, the specific dysregulated pathways in PTB remain unclear.

Increased nucleos(t)ide demand supports cellular proliferation and repair mechanisms under pathological conditions such as PTB and may help the placenta overcome stress. This highlights the need for a deeper understanding of how nucleos(t)ide metabolism and adenosine signaling pathways regulate placental function.

In this study, we investigate the dynamic regulation of nucleos(t)ide metabolism, transport, and adenosine signaling during placental development, from first trimester to term and across CTB differentiation to STB, and their dysregulation in PTB. We hypothesize that nucleos(t)ide metabolism plays a critical role in placental adaptation throughout gestation, with metabolic shifts supporting trophoblast differentiation and placental maturation. In PTB, these pathways may be further upregulated as an adaptive response to placental stress. To address this, we analyzed gene expression, metabolomic profiles, and trophoblast differentiation using human placental samples from early gestation, term, and PTB conditions, complemented by a rat model for developmental context. A more comprehensive understanding of nucleos(t)ide pathways could provide new insights into placental function and its role in pregnancy complications, including preeclampsia, fetal growth restriction, and PTB, potentially informing therapeutic interventions.

## Materials and methods

### Collection of human placentas

Human placental samples were obtained from two distinct cohorts, analyzed in two separate comparisons: first trimester vs. term placentas to study placental development and PTB vs. term placentas to investigate metabolic alterations under pathological conditions. For the developmental study, first-trimester (*n* = 10) and term placentas (*n* = 10) were analyzed. A separate set of term placentas (*n* = 10) was used for the PTB vs. term comparison, ensuring that developmental analysis was independent of pathological conditions. PTB placentas (*n* = 10) were selected from spontaneous preterm births without infection-related etiology and matched to term samples by gestation age where possible. First-trimester and term samples for the developmental study were obtained from a previously characterized cohort (Karahoda et al.^24^), while PTB and term samples for the PTB study were sourced from a separate cohort (Cifkova et al.^23^). Only placentas from singleton pregnancies with no maternal metabolic disorders, infections, or known chromosomal abnormalities were included. Detailed patient characteristics are provided in Supplementary Tables 1 and 2. All placentas were collected at the Department of Obstetrics and Gynecology, University Hospital in Hradec Kralove, Czech Republic, between May 2019 and May 2022. Informed written consent was obtained from all participants, and the study was approved by the University Hospital Research Ethics Committee (201006 S15P). All methods involving human samples were performed in accordance with the Declaration of Helsinki and relevant institutional guidelines and regulations.

## Analysis of purine and pyrimidine metabolites in the placenta

Sample preparation and the analysis of purine and pyrimidine metabolite content were carried out in the previous study^23^. Placenta samples were analyzed using the UHPLC/MS with an Acquity I-class UPLC instrument and a Vion IMS QTOF mass spectrometer (Waters, Milford, MA, USA). An Acquity UPLC HSS T3 column (150 × 2.1 mm, 1.8 μm, Waters) and gradient of 0.1% formic acid in water and 0.1% formic acid in methanol was used for separation. For additional details, see^23^.

## BeWo cell line cultivation

BeWo cells (human choriocarcinoma-derived cell line) were obtained from the European Cell Culture Collection (United Kingdom). BeWo cells were cultured in Ham F-12 medium (Sigma Aldrich St. Louis, MO, USA, N6658) supplemented with 10% fetal bovine serum (FBS, Capricorn Scientific GmbH, Ebsdorfergrund, Germany, 10-FBS-11 F). To induce differentiation to mimic the STB stage, the BeWo cell line was subjected to forskolin (FSK, MedChemExpress LLC, USA, HY-15371) used at a concentration of 50 µM for 24 h and since FSK is soluble in dimethyl sulfoxide (DMSO, Sigma Aldrich, Aldrich St. Louis, MO, USA, D8418) we treated BeWo cells with 0.05% DMSO as a control^25^. To confirm the differentiation process, hCG secretion by BeWo cells at the CTB stage and FSK-treated BeWo cells at the STB stage was measured in the cell culture medium^26^ and by analyzing the gene expression of *ERVW-1*. All cells were cultured at 37 °C, 5% CO_2_ with daily changes of the medium. The experiments were conducted with three biological replicates for primary cells and five biological replicates for BeWo cells, each analyzed in technical triplicates.

## Primary cells of the human trophoblast isolation

Human-term placenta primary trophoblast cells were isolated using three steps of enzymatic digestion and Percoll gradient separation, as previously described^24,26^. Approximately 40–50 g of villous tissue was dissected from the maternal decidua and extensively washed in sterile 0.9% NaCl to remove blood. The tissue was finely minced and subjected to three consecutive enzymatic digestions at 37 °C for 20 min each using a digestion buffer containing 0.25% trypsin and 300 IU/mL DNase I (both from Sigma–Aldrich). Following each digestion, the supernatant containing dissociated cells was filtered through sterile gauze and pooled. The resulting cell suspension was layered over FBS and centrifuged at 1,000 × g for 15 min 10 °C. The cell pellet was resuspended in DMEM (high glucose, GlutaMAX, Capricorn Scientific GmbH, Ebsdorfergrund, Germany) and passed through a 100 μm cell strainer. For enrichment of CTBs, a discontinuous Percoll density gradient (35–50%) was used. After centrifugation, the CTB fraction was collected from the interphase corresponding to a density of 1.046–1.065 g/mL. Cells were cultured in DMEM (high glucose, GlutaMAX) supplemented with 10% FBS, penicillin 100 U/ml, and streptomycin 0.1 mg/ml. Cells were seeded for 8 h to reach the CTB stage and the STB stage is obtained by spontaneous fusion over 72 h in culture (with a daily change of medium). To confirm the differentiation process in primary cells, hCG secretion by CTB and STB cells was measured in the cell culture medium^27^.

The purity of isolated cells was assessed by flow cytometry following dual staining with directly labeled antibodies against epithelial and non-epithelial markers. Cells cultured on CellBIND^®^ plates were detached using Accutase^®^, fixed in 4% formaldehyde, and permeabilized with 0.5% Tween-20 in phosphate-buffered saline. Staining was performed in a buffer containing 5% FBS and 0.1% Tween-20 using two antibody cocktails: (1) CK-7 (AF488^®^) with vimentin (AF647^®^), and (2) E-cadherin (AF488^®^) with von Willebrand factor (AF647^®^). After incubation and washing, cells were analyzed on a SA3800 Spectral Cell Analyzer (Sony Biotechnology, San Jose, CA, USA) flow cytometer, with data collected from at least 10,000 events per sample. On average, 94.18% of the isolated cells were CK-7 positive, 3.95% were vimentin positive, and less than 0.1% were positive for either von Willebrand factor or E-cadherin, confirming high purity of the trophoblast preparations.

## Collection of rat placentas

Pregnant Wistar rats (12–18 weeks of age) were obtained from MediTox s.r.o (Czech Republic) and housed under standard laboratory conditions, 22 ± 2 °C, 12:12 light/dark cycle, food Altromin (1324, Altromin, Germany) and water *ad libitum*. GD1 was established following the detection of sperm in vaginal swabs after overnight mating. Placentas were collected at GD12, GD15, and GD20 (*n* = 10 per GD), with samples obtained from five independent dams (two placentas per dam: one male and one female). At GD20, placentas were sexed based on fetal gonadal morphology to assess potential sex differences. At defined GDs, pregnant rats were anesthetized with an intravenous bolus of 40 mg/kg pentobarbital, and after induction of deep anesthesia, the uterus was exposed, placentas were excised rapidly within 1–2 min, and tissues were immediately snap-frozen in liquid nitrogen. Potassium chloride (150 mg/kg) was administered to euthanize the animals after the surgery. For gene expression analyses, values from the male and female placenta within each dam were averaged, yielding a final *n* = 5 per GD. This approach ensured that inter-dam variability was accounted for while maintaining statistical robustness. Detailed characteristics of the animals are provided in Supplementary Table 3. Gestation time points were selected to capture key developmental milestones: GD12, aligns with 4–6 weeks of human embryonic development and marks the initial formation of the labyrinth zone; GD15, the definitive placenta begins to form, and the basal zone is fully developed; GD20, placental growth reaches a plateau phase in preparation^28,29^. All procedures were approved by the Ethical Committee of the Faculty of Pharmacy in Hradec Kralove and the Ministry of Education, Youth and Sports (MSMT-13975/2023-3) and followed ARRIVE guidelines. All experiments were conducted in compliance with institutional and national regulations, as well as the ARRIVE guidelines for reporting animal research. Placental tissue was dissected and used for expression analysis, with samples stored at − 80 °C until further analysis.

### RNA isolation, reverse transcription, and quantitative PCR analysis

Total RNA was extracted from human and rat placental tissues and cultured cells using Tri-Reagent solution (Molecular Research Center, Cincinnati, OH, USA), following the manufacturer’s protocol. RNA purity was assessed by A_260/A280_ and A_260/A230_ ratios using a NanoDrop™ 1000 Spectrophotometer (Thermo Fisher Scientific, USA); only samples with ratios between 1.75 and 2.2 were selected for further analysis. RNA integrity was verified by agarose gel electrophoresis. Absorbance at 260 nm was used for the calculation of the total RNA concentration. Reverse transcription was performed using 5 µg of total RNA and the iScript Advanced cDNA Synthesis Kit using T100 Thermal Cycler (Bio-Rad, Hercules, CA, USA). Quantitative PCR analysis of gene expression in human, rat placenta, and cells was performed using QuantStudio5 (Thermo Fisher Scientific, Waltham, MA, USA). cDNA (12.5 ng/µl) was amplified in a total reaction volume of 5 µL/well using the TaqMan^®^ Fast Advanced Master Mix (Thermo Fisher Scientific, Waltham, MA, USA) and predesigned TaqMan^®^Real-Time Expression PCR assays (Supplementary Tables 4 and 5). Each sample was amplified in triplicate, using the following PCR cycling profile: 95 °C for 20 s, followed by 40 cycles at 95 °C for 1 s and 60 °C for 20 s^30,31^. For additional details on sample input, assay IDs, raw Ct values, normalization strategy, and processed data, please refer to the dataset available in the GEO repository (GSE291205), as noted in the “Availability of data and material” section.

## Gene categorization for the analysis

We grouped genes according to the pathways they are involved in, including the purine and pyrimidine *de novo* and salvage pathways, and the uncategorized group for genes involved in both purine and pyrimidine *de novo* pathways (Supplementary Fig. 1 and 2), as well as nucleoside transporters^4,32^. Furthermore, we created a separate group for adenosine metabolism, as adenosine has multiple roles in the body, including its function as a signaling molecule through adenosine receptors^33,34^. The genes belonging to the individual groups are listed in Supplementary Tables 4 and 5.

## Data processing and statistical analysis

The data were analyzed using R statistical software^35^ and functions integrated with *tidyverse* package^36^. The graphs were created using the packages *ggplot2*, *ggbeeswarm*, *ggpubr*, *ggprism*, *ggrepel*, and *ggtext*^37–43^. The statistical evaluation was performed by R stat functions or the *rstatix* package^44^. The gene expression data were processed on the principle of delta-delta Ct method^45^. The interplate calibrator reaction was analyzed in each run in triplicates and the mean value was subtracted from Ct values in all analyses and the *dCt* values were generated. For the normalization mean of the *dCt* values of reference gene expression of *B2M*, *GAPDH*, and *YWHAZ* was used. The normalized *dCt*_*NORM*_ values were acquired by subtraction of *dCt* value of the target gene from the *dCt* value of the reference gene. These values were re-scaled to the median of the reference group and presented as log2 of expression corresponding to a difference between *dCt*_*NORM*_ of the sample and the median of *dCt*_*NORM*_ values of the reference group. The log2 of expression values between sample groups were evaluated with a non-parametric two-sample Wilcoxon rank-sum test (Mann-Whitney). The non-parametric Kruskal-Wallis and Dunn’s post-hoc test was used for evaluations between more than two groups. The statistical tests implemented in the R package *rstatix* were employed^44^. The Pearson correlation coefficients for gene-to-metabolite and gene-to-gene relationships were computed using gene expression data obtained from 10 PTB placentas and 10 term placentas quantified in this study and metabolite levels previously determined by Cifkova et al.^23^. Thus, the total sample size for these correlations amounted to 20. The correlation factors and p-values were calculated using *rstatix* functions and the results were plotted by *ggplot2* package^36,37,40^. In our study, a Pearson correlation value ranging from (0.5 to 0.7 or −0.5 to −0.7) indicates a moderate correlation whether positive or negative, while a positive or negative correlation from (−0.7 to −0.9, 0.7 to 0.9) indicates a strong correlation.

## Results

### Gene expression dynamics of nucleos(t)ide pathways in first-trimester and term placentas

**Fig. 1:**
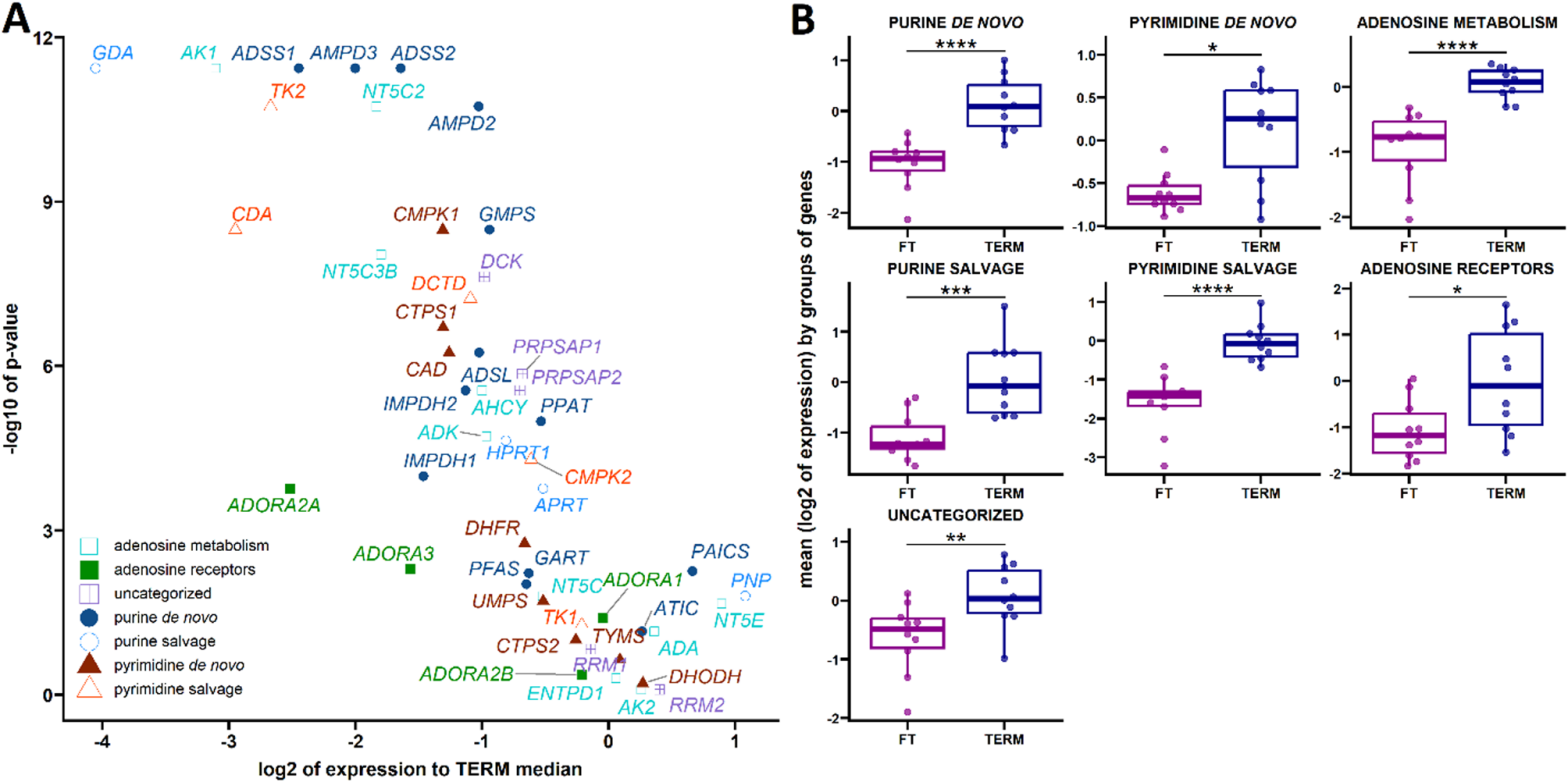
Comparative Analysis of Gene Expression in First-Trimester Placentas Compared to Term Placentas. Volcano plots display genes when comparing first-trimester (FT) to term placentas (**A**). Box plots illustrate differences between FT and term placentas (**B**). Log2 expression values for all genes in each gene group were averaged and presented as mean log2 expression values relative to the term group. Statistical analysis was performed using the Wilcoxon rank-sum test (*p < 0.05; **p < 0.01; ***p < 0.001; ****p < 0.0001 relative to the term).

In the initial phase of our study, we focused on changes in gene expression profiles between the first-trimester and term human placenta. Using the Wilcoxon signed-rank test, we found that levels of genes encoding proteins involved in nucleos(t)ide homeostasis are lower in the first-trimester placenta compared to the term placenta (Fig. 1A and Supplementary Fig. 3), all defined groups were decreased in the first-trimester placenta (Fig. 1B). Specifically, adenosine receptors (*ADORA2A*), adenosine metabolism (*ADK*, *AHCY*, *AK1*, *NT5C2*, and *NT5C3B*), purine *de novo* synthesis (*ADSL*, *ADSS1*, *ADSS2*, *AMPD2*, *AMPD3*, *GMPS*, *IMPDH1*, *IMPDH2*, and *PPAT*) (Fig. 1A and Supplementary Fig. 3A, 3B, and 3D) were downregulated, while *CAD*, *CMPK1* and *CTPS1* were downregulated in pyrimidine *de novo* synthesis (Fig. 1A and Supplementary Fig. 3F). In the purine salvage pathway, we observed downregulation of *APRT*, *GDA*, and *HPRT1* (Fig. 1A and Supplementary Fig. 3E), and in the pyrimidine salvage pathway, *CDA*, *CMPK2*, *DCTD*, and *TK2* were downregulated in the first-trimester placenta (Fig. 1A and Supplementary Fig. 3G). In the uncategorized genes, *DCK*, *PRPSAP1*, and *PRPSAP2* were also downregulated in the first-trimester placenta (see Fig. 1A and Supplementary Fig. 3C). When examining potential sexual dimorphism, no differences in expression profiles were observed between term placentas from male (*n* = 5) and female (*n* = 5) fetuses (data not shown).

### Regulation of nucleos(t)ide and adenosine pathways in preterm birth placentas

**Fig. 2:**
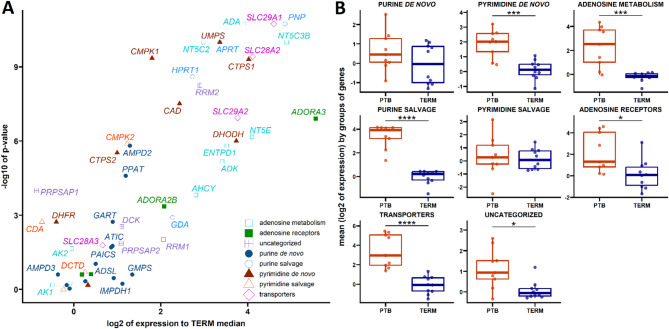
Comparative Analysis of Gene Expression in PTB Placentas Compared to Term Placentas. Volcano plots display genes exhibiting pronounced and significant changes when comparing term placentas to preterm (PTB) placentas (**A**). Box plots illustrate differences between term and PTB placentas (**B**) (*p < 0.05; ***p < 0.001; ****p < 0.0001, relative to the term).

In the following step, we compared the levels of the same set of genes in PTB and term placentas. All defined groups showed increased gene expression levels in the PTB placenta except for the purine *de novo* and pyrimidine salvage pathways (Fig. 2B). We observed upregulation in adenosine receptors (*ADORA2B* and *ADORA3*), adenosine metabolism-related genes (*ADA*, *ADK*, *AHCY*, *ENTPD1*, *NT5C2*, *NT5C3B*, and *NT5E*), and transporters (*SLC28A2*, *SLC29A1*, and *SLC29A2*) (Fig. 2A and Supplementary Fig. 4A, 4B, and 4H). Similarly, we observed upregulation in genes linked to the pyrimidine *de novo* pathway (*CAD*, *CMPK1*, *CTPS1*, *CTPS2*, *DHODH*, and *UMPS*) (Fig. 2A and Supplementary Fig. 4F) and the pyrimidine salvage pathway (*CMPK2*) (Fig. 2A and Supplementary Fig. 4G). In the purine *de novo* and salvage pathways, we observed elevated levels of *AMPD2*  and *PPAT* (Fig. 2A and Supplementary Fig. 4D) and *APRT*, *HPRT1*, and *PNP* (Fig. 2A and Supplementary Fig. 4E), respectively, in the PTB placenta. In the group of uncategorized genes, *PRPSAP1* was downregulated, but *RRM2* was upregulated in the PTB placenta (Fig. 2A and Supplementary Fig. 4C).

### Gene-metabolite and gene-gene correlations in placental nucleos(t)ide metabolism

**Fig. 3:**
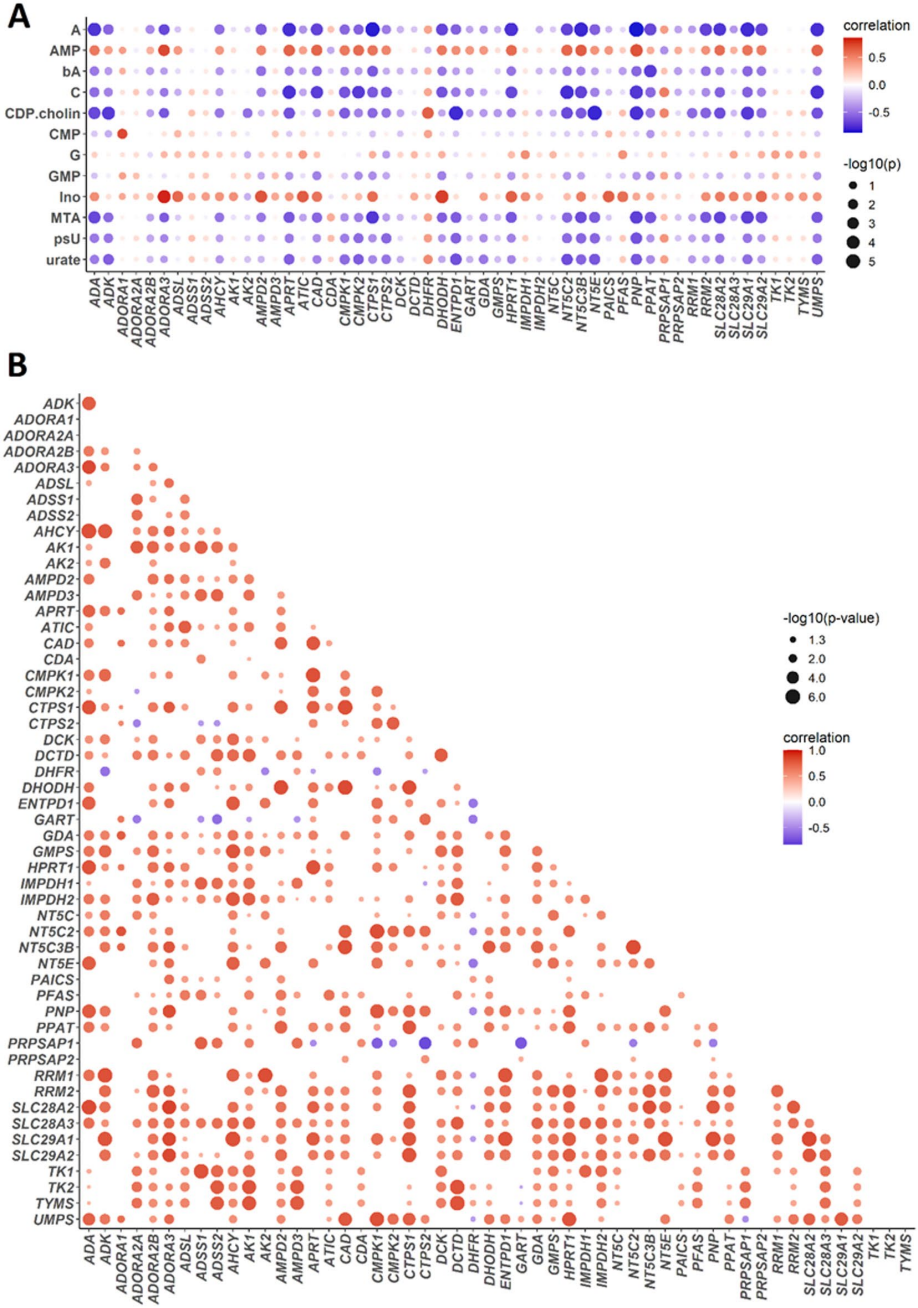
Gene-to-nucleos(t)ide metabolite and gene-to-gene correlations in the placenta. Bubble plot representing the correlations between genes and metabolites (**A**) and gene-to-gene correlations (**B**) in both PTB and term placentas. The color indicates the value of the correlation coefficient calculated by Pearson correlation analysis (blue for negative correlations and red for positive correlations). The size of the circle represents the -log10 p-value, with a threshold >1 in subFig A and >1.30 in subFig B, indicating statistical significance. The raw data are available here^47^. Abbreviations for subFig A: A (adenosine), AMP (adenosine monophosphate), bA (base adenine), C (cytidine), CDP-choline (cytidine diphosphocholine), CMP (cytidine monophosphate), G (guanosine), GMP (guanosine monophosphate), Ino (inosine), MTA (methylthioadenosine), psU (pseudouridine).

In our previous study, we identified dysregulated metabolite levels, including adenosine, adenine, AMP, inosine, guanosine, and cytidine, in PTB placentas^23^. To explore the link between these metabolite levels and gene expression changes we performed a Pearson correlation analysis and revealed moderate to strong negative correlations between adenosine levels and genes involved in purine salvage, purine *de novo* synthesis, and adenosine metabolism. Notably, *PNP* levels (*r* = −0.87, -log10p = 5.876), *ADA* (*r* = −0.80, -log10p = 4.334), *SLC29A1* (*r* = −0.83, -log10p = 5.0), *HPRT1* (*r* = −0.81, -log10p = 4.346), *PPAT* (*r* = −0.73, -log10p = 3.444), *ADORA3* (*r* = −0.73, -log10p = 2.514), *NT5C2* (*r* = −0.75, -log10p = 3.511), *NT5C3B* (*r* = −0.84, -log10p = 4.954), *NT5E* (*r* = −0.64, -log10p = 2.498), *ADK* (*r* = −0.61, -log10p = 2.288) and *AMPD2* (*r* = −0.65, -log10p = 2.619). For adenine, we identified moderate negative correlations with *PNP* (*r* = −0.61, -log10p = 2.244), *CTPS1* (*r* = −0.66, -log10p = 2.559), *SLC29A1* (*r* = −0.61, -log10p = 2.235). AMP levels, in contrast, showed positive correlations with *APRT* (r = + 0.69, -log10p = 2.847), and *ADSL* (r = + 0.46, -log10p = 1.333). Similarly, inosine levels were positively correlated with genes involved in purine *de novo* synthesis, salvage pathways, and adenosine metabolism such as *PAICS* (r = + 0.70, -log10p = 3.088), *ATIC* (r = + 0.71, -log10p = 3.043), *HPRT1* (r = + 0.70, -log10p = 2.931), *ADA* (r = + 0.51, -log10p = 1.580). Cytidine levels also predominantly negatively correlated with genes in pyrimidine pathways, like *CAD* (*r* = −0.77, log10p = 3.677) and *UMPS* (*r* = −0.81, -log10p = 4.376). Interestingly, there was also a correlation between inosine and pyrimidine pathways; *CAD* (r = + 0.62, -log10p = 2.189), *DHODH* (r = + 0.81, -log10p = 4.101), Guanosine exhibited a moderate positive correlation with *IMPDH1* (r = + 0.5, p-value = 1.494), while guanosine monophosphate (GMP) negatively correlated with *PPAT* (*r* = −0.46, p-value = 1.347) (Fig. 3A). Nucleos(t)ide metabolism is reportedly tightly controlled by transcriptional, post-transcriptional, and feedback inhibition mechanisms^46^therefore we performed gene-to-gene correlation analysis. It revealed predominantly strong positive associations, with the exception of *PRPSAP1* with *PNP* (*r* = −0.60, -log10p = 1,995), *UMPS* (*r* = −0.52, -log10p = 1.407), *CMPK1* (*r* = −0.74, -log10p = 3.161), *CMPK2* (*r* = −0.60, -log10p = 1,879), and *NT5C2* (*r* = −0.60, -log10p = 1.853), as well as *PNP* with *DHFR* (*r* = −0.55, -log10p = 1.835) (Fig. 3B).

### Developmental and sex-specific gene expression dynamics in rat placentas during gestation

**Fig. 4:**
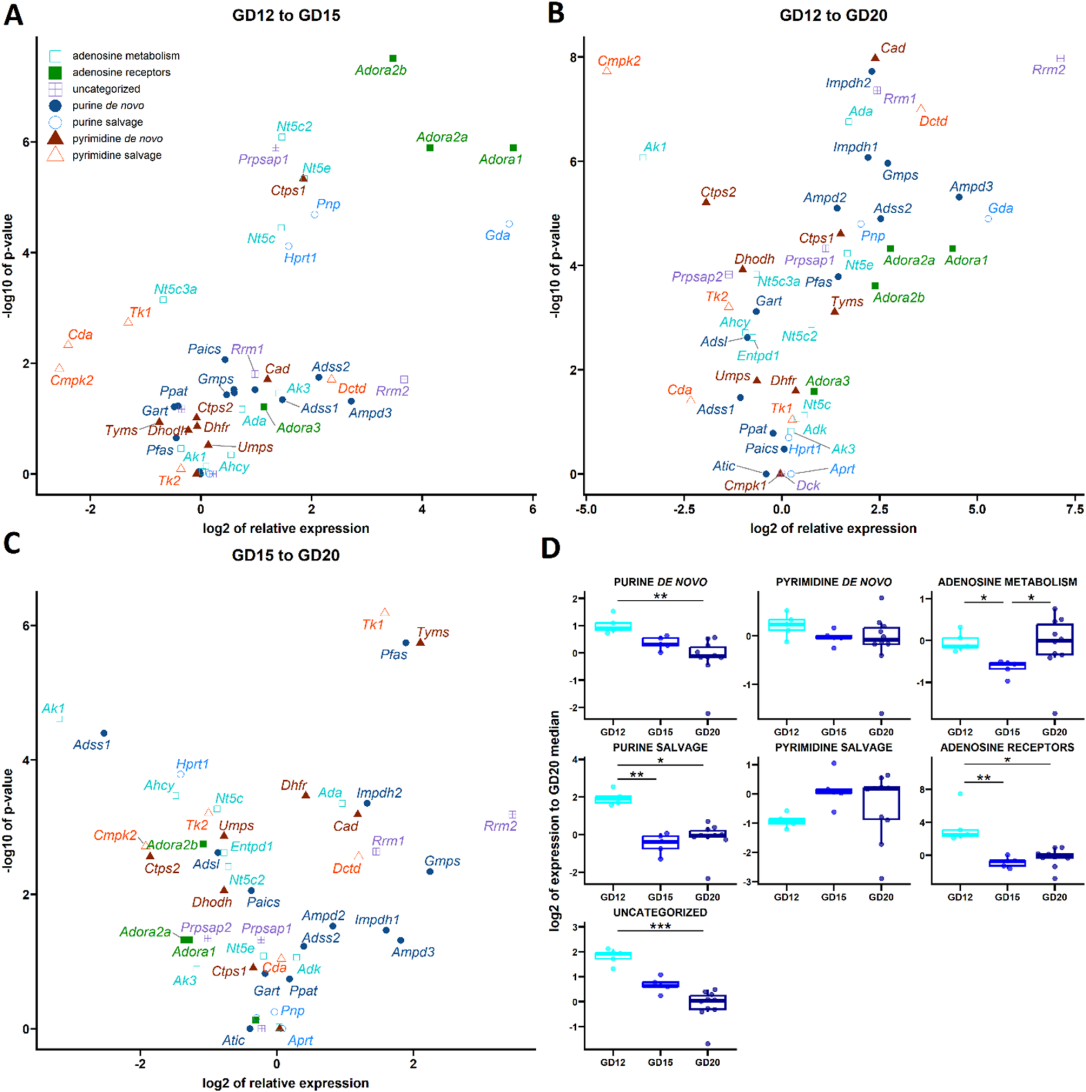
Comparative analysis of gene expression in the rat placenta from midgestation to term. Volcano plots display genes exhibiting significant changes in comparisons from GD12 to GD15 (**A**), GD12 to GD20 (**B**), and GD15 to GD20 (**C**). Box plots illustrate differences in gene expression (**D**). Log2 expression values for all genes in each gene group were averaged and presented as mean log2 expression values relative GD20. Statistical analysis was performed using the Kruskal–Wallis test and Dunn’s post-hoc test (*p < 0.05; **p < 0.01; ***p < 0.001; relative to the GD20).

We collected rat placentas at GD12, 15, and 20, covering the period of extensive growth^28^ that corresponds to early second-trimester development in human placentas^29,48^ to further study the developmental regulation of nucleos(t)ide metabolism. Genes involved in the purine *de novo* pathway showed significant upregulation at GD12 compared to GD20, while genes associated with the purine salvage pathway showed higher expression at GD12 relative to both GD15 and GD20. Additionally, gene expression levels for enzymes related to adenosine metabolism were reduced at GD15 in comparison with GD12 and GD20. Expression levels of adenosine receptors were higher at GD12 compared to both GD15 and GD20. For a group of uncategorized genes, expression levels were similar at GD12 and GD15 but declined at GD20. In contrast, genes associated with the pyrimidine *de novo* and salvage pathways demonstrated stable expression throughout the gestation periods examined (Fig. 4D).

Further analysis of gene expression between GD12 and GD15 showed that adenosine receptor genes (*Adora1*, *Adora2a*, *Adora2b*), adenosine metabolism genes (*Nt5c*, *Nt5c2*, *Nt5e*), and genes in the purine salvage pathway (*Gda*, *Hprt1*, *Pnp*) were upregulated at GD12, while *Nt5c3a* within the adenosine metabolism pathway was downregulated (Fig. 4A, 4D and Supplementary Figs. 5A, 5B, and 5E). Expression of genes across the pyrimidine *de novo*, salvage, and purine pathways, as well as uncategorized genes, remained largely stable; however, *Ctps1* (pyrimidine *de novo*) and *Prpsap1* (uncategorized) were notably upregulated at GD12 (Fig. 4A, 4D and Supplementary Fig. 5C and 5F).

When comparing GD12 and GD20, we observed elevated expression at GD12 in genes related to the purine *de novo* and salvage pathways, adenosine receptors, and uncategorized genes (Fig. 4D). Specifically, adenosine receptor genes (*Adora1*, *Adora2a*, *Adora2b*), along with adenosine metabolism genes (*Ada*, *Nt5e*), were upregulated at GD12, whereas *Ak1* and *Nt5c3a* were downregulated (Fig. 4B, Supplementary Fig 5A, and 5B). Genes involved in the purine *de novo* pathway, including *Adss2*, *Ampd2*, *Ampd3*, *Gmps*, *Imphd1*, *Impdh2*, and *Pfas*, were upregulated at GD12, with the exception of *Gart*, which was downregulated (Fig. 4B and Supplementary Fig. 5D). Additionally, in the purine salvage pathway, *Gda* and *Pnp* were upregulated (Fig. 4B and Supplementary Fig. 5E). Within the pyrimidine *de novo* pathway, genes such as *Cad*, *Ctps1*, and *Tyms* were upregulated, while *Ctps2* and *Dhodh* were downregulated (Fig. 4B and Supplementary Fig. 5F). In the pyrimidine salvage pathway, *Dctd* was upregulated, while *Cmpk2* and *Tk2* were downregulated (Fig. 4B and Supplementary Fig. 5G). Among uncategorized genes, *Prpsap1*, *Rrm1*, and *Rrm2* were upregulated, while *Prpsap2* was downregulated at GD12 (Fig. 4B and Supplementary Fig. 5C).

When comparing GD15 and GD20, there was an upregulation of *Ada*, involved in adenosine metabolism, at GD15, while *Ahcy*, *Ak1*, and *Nt5c* were downregulated (Fig. 4C and Supplementary Fig. 5B). In the purine *de novo* pathway, *Impdh2* and *Pfas* were upregulated at GD15, whereas *Adss1* was downregulated (Fig. 4C and Supplementary Fig. 5D), and *Hprt1* in the purine salvage pathway was also downregulated (Fig. 4C and Supplementary Fig. 5E). In the pyrimidine *de novo* pathway, *Cad*, *Dhfr*, and *Tyms* were upregulated (Fig. 4C and Supplementary Fig. 5F), while *Tk1* was upregulated in the pyrimidine salvage pathway, except for *Tk2*, which was downregulated (Fig. 4C and Supplementary Fig. 5G). In the uncategorized gene group, *Rrm2* was upregulated at GD15 (Fig. 4C and Supplementary Fig. 5C).

Finally, analysis of potential sexual dimorphism showed that genes such as *Adss1*, *Adss2*, *Ak3*, *Aprt*, *Atic*, *Cda*, *Cmpk1*, *Dctd*, *Paics*, *Prpsap2*, and *Tk2* were upregulated in female placentas compared to male placentas (*n* = 5 each) at GD20 (Supplementary Fig. 6).

### Gene expression changes during trophoblast differentiation and adenosine metabolism dynamics

**Fig. 5:**
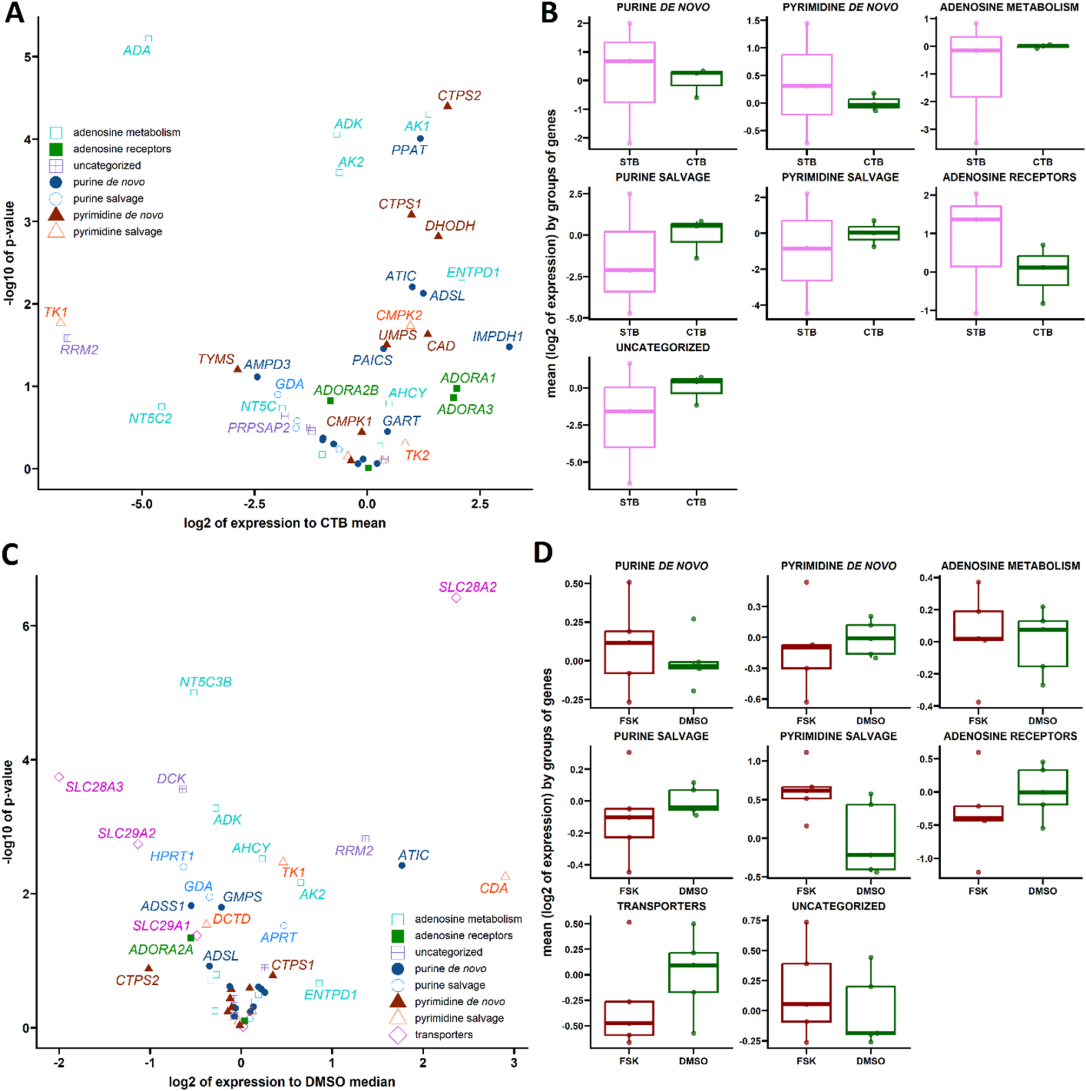
Comparison of CTB and STB stages of trophoblast. Volcano plots display genes with significant expression changes when comparing primary cell CTB to STB (**A**). Box plots show expression differences between primary CTB and STB (**B**). Volcano plots illustrate significant gene expression changes between DMSO (0.05%) and FSK (50 µM)-treated BeWo cells (**C**), and box plots depict differences between DMSO and FSK (50 µM)-treated BeWo cells (**D**). Data are presented as log2 expression values relative to the mean of CTB and DMSO. Statistical analysis was performed using the unpaired Student’s t-test, with no significant differences detected.

Consistent with our hypothesis predicting gene expression shifts from stem cell-like CTB to fused, differentiated STB, we analyzed gene expression profiles in freshly isolated CTBs and after three days of spontaneous differentiation into STBs. Our results showed no significant changes in the expression of gene groups, indicating a preserved capacity for nucleotide maintenance and signaling. However, individual gene analysis showed *AK1* upregulation and *ADA*, *ADK*, and *AK2* downregulation in STB, suggesting dynamic alterations during differentiation. Genes in the purine *de novo* synthesis pathway, such as *PPAT*, and in the pyrimidine *de novo* synthesis pathway, including *CTPS1* and *CTPS2*, were upregulated in STB (Fig. 5A and 5B, and Supplementary Fig. 7). Given the non-proliferative nature of primary CTBs, we extended our analysis to the BeWo cell line, where no group-level changes were observed. However, single-gene analyses showed *ADK* and *NT5C3B* downregulation in differentiated BeWo cells (Fig. 5C, 5D, and Supplementary Fig. 8), indicating dynamic changes in adenosine metabolism. *DCK* expression was also reduced in the uncategorized gene group in differentiated BeWo cells (Fig. 5C and Supplementary Fig. 8G). In group of nucleoside transporters, differentiated BeWo cells showed increased *SLC28A2* expression, while *SLC28A3* was downregulated (Fig. 5C and Supplementary Fig. 8H). Furthermore, *ADORA3* gene expression was absent in BeWo cells (Fig. 5C and Supplementary Fig. 8A).

### Comparative analysis of gene expressions during CTB-STB transition in trophoblast models

**Fig. 6:**
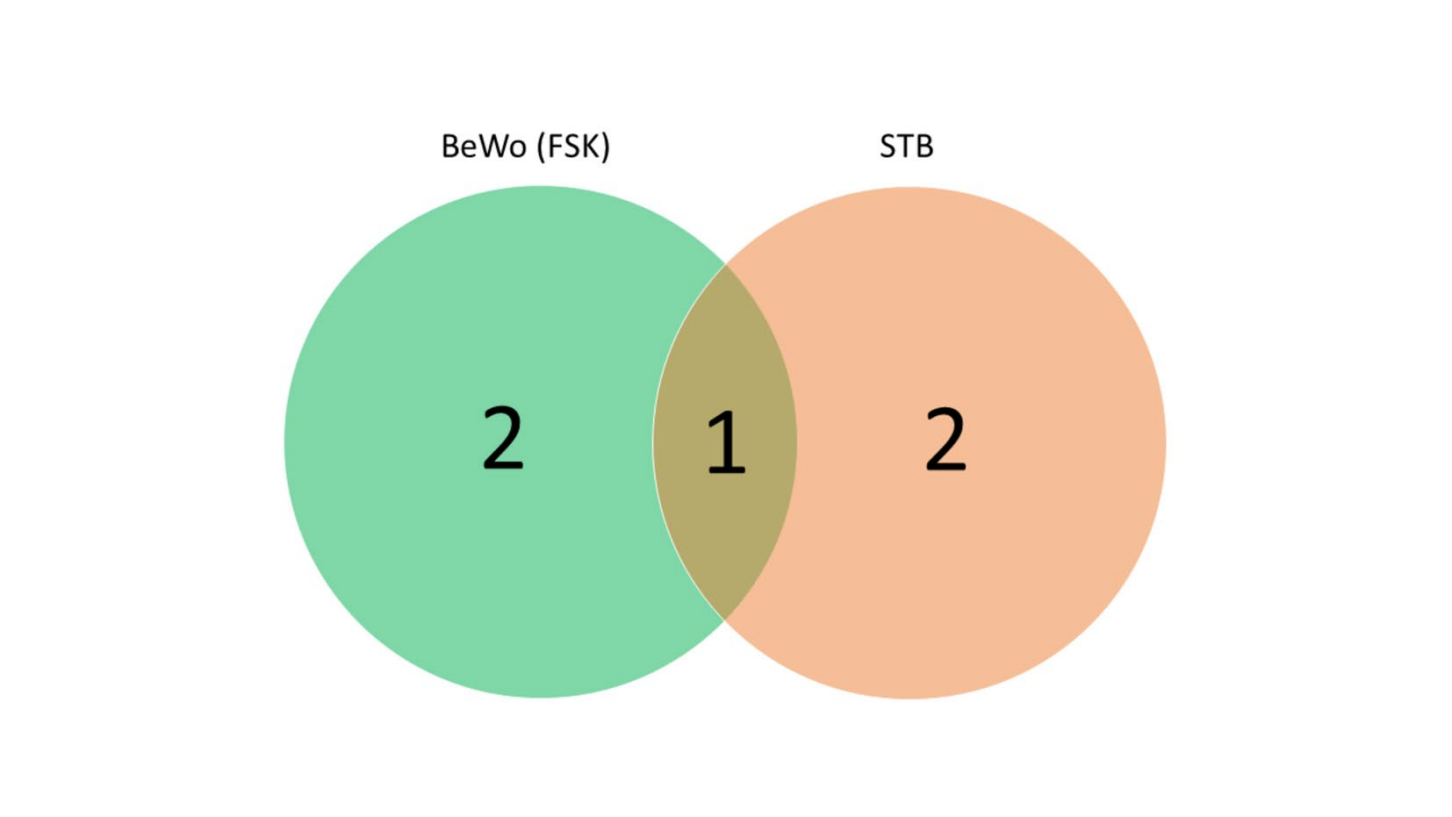
Venn diagram analysis of gene expression. This analysis focuses on downregulated genes in primary STB and forskolin-treated (50 µM) BeWo cells. In forskolin-treated BeWo cells, the downregulated genes include *ADK*, *NT5C3B*, and *DCK*. In primary STB, the downregulated genes are *ADA*, *ADK*, and *AK2*. Notably, *ADK* is the only gene commonly downregulated in both forskolin-treated BeWo cells and primary STB.

To examine expression pattern shifts between BeWo and primary trophoblast cells during the CTB-STB transition, we used Venn diagram analysis. Since differentiated BeWo cells did not show increases in metabolic pathways, we focused on genes with reduced expression. Three genes were downregulated in both primary STB and differentiated BeWo cells, with one overlapping gene, *ADK* (Fig. 6), suggesting a reduced capacity of STB to convert adenosine to adenosine monophosphate.

## Discussion

Building on prior research into nucleos(t)ide metabolism in cancer^4,8^ and the proposed significance of nucleosides for placental and fetal development^7,21–23^this study addresses gaps in understanding placental nucleos(t)ide homeostasis and adenosine signaling during gestation, trophoblast differentiation, and the pathological condition of PTB. Our findings reveal dynamic changes in these pathways, suggesting an important role in placental adaptation to metabolic demands and PTB placentas.

The placenta undergoes continuous growth throughout pregnancy^49^requiring dynamic metabolic adaptations^3,50^. Our results demonstrate a global upregulation of nucleos(t)ide metabolism from early gestation to term, likely supporting the increased biosynthetic needs of trophoblast differentiation and placental maturation.

Gene expression of key enzymes involved in adenosine production was elevated, whereas enzymes regulating adenosine degradation remained stable (Fig. 1 and Supplementary Fig. 3I). This imbalance likely contributes to the reported increase in plasma adenosine levels in term pregnancies^18^ as well as the observed adenosine high content in placental tissue^19^. These findings suggest a regulatory role for adenosine in fetal-maternal communication and placental homeostasis.

Our data indicate that both *de novo* synthesis and salvage pathways contribute comparably to nucleotide metabolism across gestation, aligning with previous work^51^. This contrasts with the preferential use of *de novo* nucleotide synthesis in proliferating tissues and salvage pathways in terminally differentiated tissues^4,52^. Strong gene-metabolite correlations (Fig. 3B) highlight the interconnected nature of adenosine metabolism, purine salvage, and transport mechanisms, suggesting that disruptions in one pathway may trigger compensatory metabolic shifts.

In PTB placentas, we identified an upregulation of genes associated with nucleos(t)ide homeostasis, adenosine metabolism, and related receptors, exceeding levels observed at term (Fig. 2 and Supplementary Fig. 4). This suggests that PTB placentas may undergo metabolic shifts in response to altered energy and biosynthetic requirements^23^. Although both adenosine production and degradation pathways are upregulated in PTB placentas (Supplementary Fig. 4J), adenosine levels remain lower than at term, suggesting a shift favoring adenosine breakdown.

This is further supported by gene-to-metabolite correlations (Fig. 3), which reveal an inverse relationship between adenosine levels and the expression of key metabolic enzymes and uptake transporters upregulated in PTB placentas (Fig. 2 and Supplementary Fig. 4B and 4H). Notably, the observed increase in *SLC29A* transporter expression^2^ was unexpected, given prior findings suggesting its constitutive expression^30^. We speculate that elevated *SLC29A* levels enhance adenosine uptake, effectively counteracting the increased extracellular adenosine production driven by NT5E upregulation. It is likely that once taken up, adenosine is metabolized by the upregulated ADA and ADK, leading to increased inosine and AMP production in PTB placentas, both of which correlate positively with the expression of their respective metabolic genes. This metabolic shift favoring downstream adenosine metabolites was further confirmed at the metabolite level (Supplementary Fig. 9). Given the roles of inosine and AMP in cellular metabolism, stress adaptation, and organ protection, particularly under ischemic and inflammatory conditions, their accumulation in PTB placentas likely reflects an adaptive mechanism to sustain nucleotide availability under stress rather than a cause of the pathology. In fact, this shift may help counteract the reduced extracellular adenosine levels and support continued placental function despite metabolic challenges^53,54^.

Elevated levels of *ADORA2B*, *ADORA3*, *NT5E*, *ADA*, and *AHCY* in PTB placentas are consistent with findings from preeclamptic placentas^20,55–57^. Increased *AHCY* expression, a key enzyme in S-adenosylmethionine (SAM) synthesis, suggests altered methylation potential that may influence fetal development^58^. Additionally, elevated AMPD2 expression may reduce adenosine availability while increasing IMP levels, a compound with known anti-inflammatory properties^59^.

Upregulation of genes in the purine *de novo* pathway, such as *PPAT*^60 ^and salvage pathway genes, including *PNP*, *APRT*, and *HPRT1* suggest a shift toward increased nucleotide turnover in PTB placentas. Given that PPAT and PNP are highly expressed in aggressive cancers, their elevated levels in PTB may reflect an increased demand for nucleotides in response to stress^60,61^. Increased *APRT* expression in PTB placentas suggests a metabolic shift from energy intensive *de novo* synthesis to salvage pathways, a feature commonly observed in cancer cells^62^possibly reflecting mitochondrial disturbances in PTB placentas^63^. Similarly, HPRT may be crucial for maintaining placental immune function^64^.

Regarding pyrimidine metabolism, the upregulation of *CMPK1*, *CMPK2*, *CTPS2*, *CAD*, *DHODH*, and *UMPS* suggests a role in supporting placental growth under stress, reflecting their established functions in cancer cell proliferation^65–70^. Notably, CMPK1 inhibition reduces cancer cell proliferation^65^while CTPS2 is implicated in DNA repair, potentially aiding the placenta in maintaining genomic stability under adverse conditions^67^. CMPK2 inhibition has been found to reverse cellular senescence and promote cell differentiation via mitochondrial enhancement^71^suggesting a possible role in placental adaptation.

Additionally, overexpression of RRM2, associated with poor prognosis in prostate cancer due to its role in DNA repair and synthesis^72^and downregulation of PRPSAP1, shown to inhibit neuroblastoma and tumor growth *in vitro* and *in vivo* by disrupting DNA synthesis^73^, further underscore the relevance of these pathways (Fig. 2A and Supplementary Fig. 4). A more detailed summary of dysregulated gene functions in PTB placentas is provided in Supplementary Table 6. To ensure the specificity of our findings, we carefully dissected the placental villous region to exclude maternal decidua and basal plate, thereby minimizing contamination. Importantly, the gene expression profiles observed in third-trimester placental tissue closely align with those detected in isolated primary trophoblasts (Supplementary Fig. 3 and 7), which are widely regarded as minimally contaminated and representative of trophoblast-specific expression. This strong overlap suggests that the transcriptional signals in the bulk tissue predominantly reflect the trophoblast layer, with any contribution from other cell types such as fetal endothelial or stromal cells likely being negligible.

Because healthy human placentas from the second trimester are rarely available, we used pregnant Wistar rats as a surrogate model. Placentas were collected at GD 12, 15, and 20, stages that parallel the second trimester in humans, allowing us to examine gene-expression changes during key phases of placental maturation and to bridge the gap between our first-trimester and term human samples^28,29^. The expression patterns observed in rat placentas did not follow the same increasing trend but rather stage specific regulation. These differences are most likely due to species-specific regulation or developmental timing, not to the use of pentobarbital, as the placenta was excised and snap-frozen within ~1–2 min after anesthesia induction, a period well below the ≥5 min required to trigger stress-responsive transcription factor or kinase activation^74^ and the ≥10 min warm ischemia threshold for light-microscopic change^75^. Moreover, *in situ* dual perfusion studies using the same pentobarbital regimen preserve placental morphology and membrane transport including active ones for 60 min^76^^[,77^^[,78^^[,79^. Our study of sexual dimorphism revealed upregulation of specific genes in female placentas (Supplementary Fig. 6), suggesting potential differences in nutritional or developmental requirements between male and female fetuses. This upregulation may function as a compensatory mechanism supporting the development of smaller-sized female fetuses^80^. No significant differences were observed in human samples, possibly due to limited sample size (*n* = 5 per group), interindividual variability, or differences in gestation length.

In the final phase, we examined gene expression shifts during trophoblast differentiation using nonproliferating primary CTB and STB, as well as proliferating BeWo cells treated with forskolin^30^. Contrary to the common assumption that proliferative cells rely on *de novo* synthesis while differentiated tissues depend on salvage pathways^6^we observed only minor changes in nucleos(t)ide metabolism during the CTB to STB transition. These findings indicate that nucleos(t)ides are vital for both proliferative and differentiated states (Fig. 5C, 5D, and Supplementary Fig. 7 and 8), aligning with recent report^51^ and observation that the placenta utilizes both *de novo* and salvage pathways at comparable levels throughout pregnancy. In both models, we observed a downregulation of *ADK* (Fig. 6), a key regulatory enzyme. Moreover, the reduction of *ADA* in primary STB suggests a diminished capacity to convert adenosine to inosine, potentially resulting in increased adenosine levels. These findings further imply a potential upregulation of purine and pyrimidine synthesis, both critical for maintaining energy homeostasis and protecting tissues^81^.

Our study reveals certain limits, primarily the small sample size and lack of gestation age-matched controls^82,83^which could affect the generalizability of our findings. The bulk tissue gene expression analysis averages signals across diverse placental cell types, which may mask cell-type-specific regulatory mechanisms and introduce variability. The rat model offers insights into gene expression dynamics, yet human-rat placental differences^28,29^ may limit extrapolation of collected results. As our study focused specifically on the villous CTB–STB axis, the main trophoblast layer responsible for maternal-fetal exchange, it did not include the extravillous trophoblast lineage involved in uterine invasion. In addition, we did not perform functional assays to validate the roles of pyrimidine synthesis, purine salvage, and adenosine metabolism, which would further clarify the biological impact of these pathways in the placenta. While such experiments were beyond the scope of this study, we partially addressed this limitation through gene–metabolite correlation analysis, which supports the relevance of the observed transcriptional changes.

In conclusion, this study explores the dynamic regulation of nucleos(t)ide metabolism, transport, and adenosine signaling in human placentas across different gestation stages, during trophoblast differentiation, and under PTB conditions. Through gene expression and metabolome analyses, the study reveals that term placentas exhibit increased expression of genes involved in nucleotide synthesis, salvage pathways, and adenosine metabolism compared to the first trimester. It also shows that the interplay between genes and between genes and metabolites is strongly regulated. In PTB placentas, these pathways are upregulated even further, suggesting an adaptive response to maintain placental function under adverse conditions and explaining the changes in adenosine levels. Notably, the study suggests an equivalent need for nucleos(t)ides in CTBs and STBs, although adenosine levels are expected to be elevated. This indicates that nucleos(t)ides, and especially adenosine, play diverse roles beyond supporting placental growth. Overall, the findings shed light on the molecular pathways of nucleos(t)ide metabolism in the healthy and PTB placenta. However, whether these changes enhance placental resilience or contribute to dysfunction requires further functional investigation. This research might open avenues for further investigation into the therapeutic potential of targeting these pathways to mitigate the risks associated with PTB.

## Supplementary Information

Below is the link to the electronic supplementary material.

Supplementary Material 1

## Data Availability

The datasets for genes generated and/or analysed during the current study are available in the [GEO] repository, [https://www.ncbi.nlm.nih.gov/geo/query/acc.cgi? acc=GSE291205]. Additionally, the datasets related to gene-to-metabolite and gene-to-gene relationships generated and/or analysed during the current study are available in the [figshare] repository, [https://doi.org/10.6084/m9.figshare.28532213.v1].
