# Supplementary material for: Dynamic shifts in trophoblast nucleos(t)ide metabolism, transport, and adenosine signaling during gestation and preterm birth
Source: Sci Rep. 2025 Aug 18;15:30275. doi: 10.1038/s41598-025-16183-2 (PMC12361576; doi:10.1038/s41598-025-16183-2)
Supplement: Supplementary file 1 — Supplementary Material 1 [file 41598_2025_16183_MOESM1_ESM.pdf]

Supplementary information to:

Dynamic shifts in trophoblast nucleos(t)ide metabolism, transport, and adenosine signaling during gestation and preterm birth

Mohammed Ali[1], Mariia Adler[1], Antonin Libra[1,3], Ivan Vokral[1], Rona Karahoda[1], Eva Cifkova[2], Miroslav Lisa[2], Jakub Tomek[1], Magdalena Novotna[1], Frantisek Staud[1], Lukas Cerveny[1*]

[1]Department of Pharmacology and Toxicology, Faculty of Pharmacy in Hradec Kralove, Hradec Kralove, Charles University, Czech Republic

[2]Department of Chemistry, Faculty of Science, University of Hradec Kralove, Hradec Kralove, Czech Republic

[3]Institute of Clinical Biochemistry and Diagnostics, University Hospital, Hradec Kralove, Czech Republic

Keywords:

Nucleotide and nucleoside metabolism in placenta, adenosine receptors, preterm birth, nucleoside transporters, placental gene expression, cytotrophoblast and syncytiotrophoblast

Corresponding author:

Lukas Cerveny

E-mail: cervenyl@faf.cuni.cz

Address: Faculty of Pharmacy in Hradec Kralove, Charles University, Akademika Heyrovskeho 1203, Hradec Kralove 500 03, Czech Republic

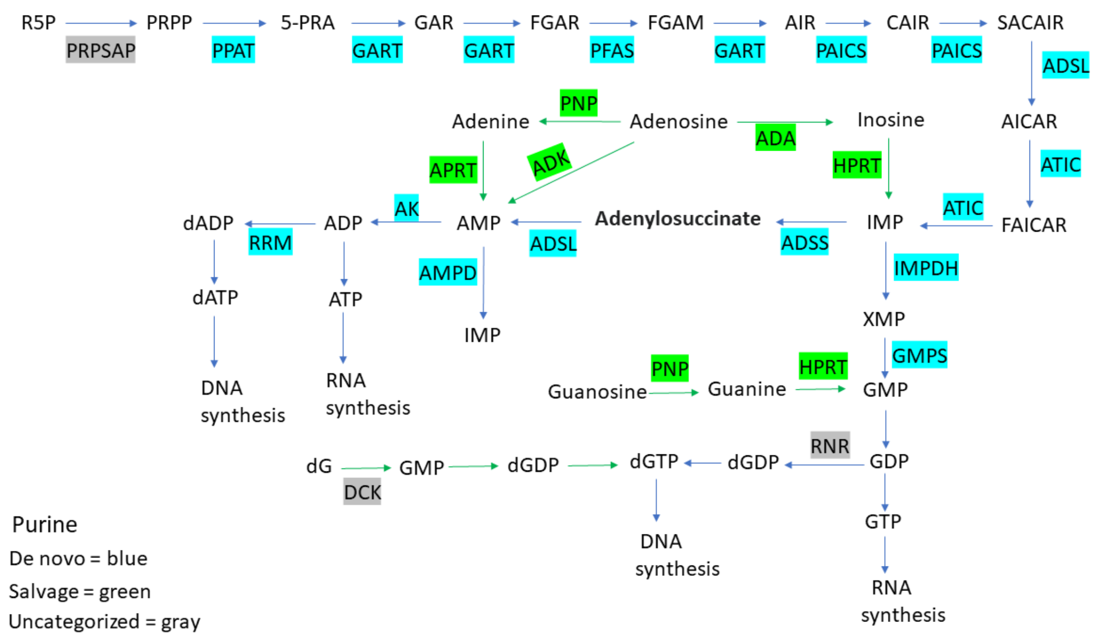

Supplementary Fig. 1. Purine *de novo* and salvage pathways (expands information provided in introduction).

The purine *de novo* synthesis is shown in blue: ribose-5-phosphate (R5P), phosphoribosyl pyrophosphate (PRPP), phosphoribosyl pyrophosphate amidotransferase (PPAT), 5-phosphoribosylamine (5-PRA), phosphoribosylglycinamide formyltransferase, phosphoribosylglycinamide synthetase, phosphoribosylaminoimidazole synthetase (GART), Glycineamide ribonucleotide (GAR), Phosphoribosyl-N-formylglycineamide (FGAR), phosphoribosylformylglycinamidine synthase (PFAS), 5-phosphoribosylformylglycinamidine (FGAM), 5-aminoimidazole ribotide (AIR), phosphoribosylaminoimidazole carboxylase (PAICS), 5-phosphoribosyl-4-carboxy-5-aminoimidazole (CAIR), phosphoribosylaminoimidazolesuccinocarboxamide (SAICAR), adenylosuccinate lyase (ADSL), 5-Aminoimidazole-4-carboxamide ribonucleotide (AICAR), 5-aminoimidazole-4-carboxamide ribonucleotide formyltransferase/IMP cyclohydrolase (ATIC), inosine monophosphate (IMP), Inosine monophosphate dehydrogenase (IMPDH), xanthosine monophosphate (XMP), guanine monophosphate synthase (GMPS), guanosine monophosphate (GMP), guanosine diphosphate (GDP), guanosine triphosphate (GTP), ribonucleotide reductase (RNR), deoxyguanosine diphosphate (dGDP), deoxyguanosine triphosphate (dGTP), adenylosuccinate synthase (ADSS), adenosine monophosphate (AMP), adenosine monophosphate deaminase (AMPD), adenosine kinase (AK), adenosine diphosphate (ADP), adenosine triphosphate (ATP), deoxyadenosine diphosphate (dADP), deoxyadenosine triphosphate (dATP). The Purine salvage synthesis is shown in green: purine nucleoside phosphorylase (PNP), adenine phosphoribosyltransferase (APRT), adenosine kinase (ADK), adenosine deaminase (ADA), hypoxanthine phosphoribosyltransferase (HPRT). The uncategorized group: phosphoribosyl pyrophosphate synthetase (PRPSAP), deoxycytidine kinase (DCK), ribonucleotide reductase (RNR).

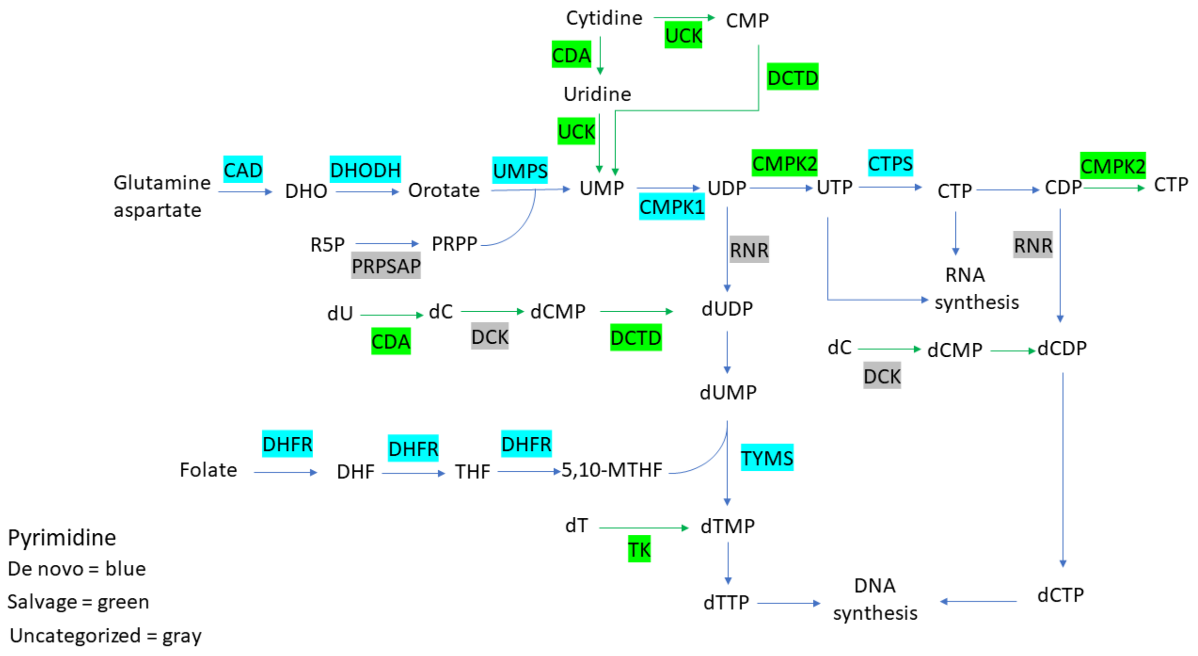

Supplementary Fig. 2. Pyrimidine *de novo* and salvage pathway (expands information provided in introduction)

The pyrimidine *de novo* synthesis is shown in blue: carbamoyl-phosphate synthetase 2, aspartate transcarbamylase, and dihydroorotase (CAD), dihydroorotate (DHO), dihydroorotate dehydrogenase (DHODH), ribose-5-phosphate (R5P), phosphoribosyl pyrophosphate (PRPP), uridine monophosphate synthetase (UMPS), uridine monophosphate (UMP), cytidine/uridine monophosphate kinase (CMPK), uridine diphosphate (UDP), uridine triphosphate (UTP), cytidine triphosphate synthase (CTPS), cytidine triphosphate (CTP), cytidine diphosphate (CDP), deoxycytidine diphosphate (dCDP), deoxycytidine triphosphate (dCTP), deoxyuridine diphosphate (dUDP), deoxyuridine monophosphate (dUMP), thymidylate synthase (TYMS), deoxythymidine monophosphate (dTMP), deoxythymidine triphosphate (dTTP), dihydrofolate reductase (DHFR), dihydrofolate (DHF), tetrahydrofolate (THF), 5,10-methylenetetrahydrofolate (5,10-MTHF). The Pyrimidine salvage synthesis is shown in (green): cytidine deaminase (CDA), uridine cytidine kinase (UCK), cytidine monophosphate (CMP), deoxycytidylate deaminase (DCTD), deoxyuridine (dU), cytidine deaminase (CDA), deoxycytidine (dC), deoxycytidine monophosphate (dCMP), deoxythymidine (dT), thymidine kinase (TK). The group of uncategorized genes: phosphoribosyl pyrophosphate synthetase (PRPSAP), deoxycytidine kinase (DCK), ribonucleotide reductase (RNR).

Comparison of gene expression between first-trimester (FT) and term placentas

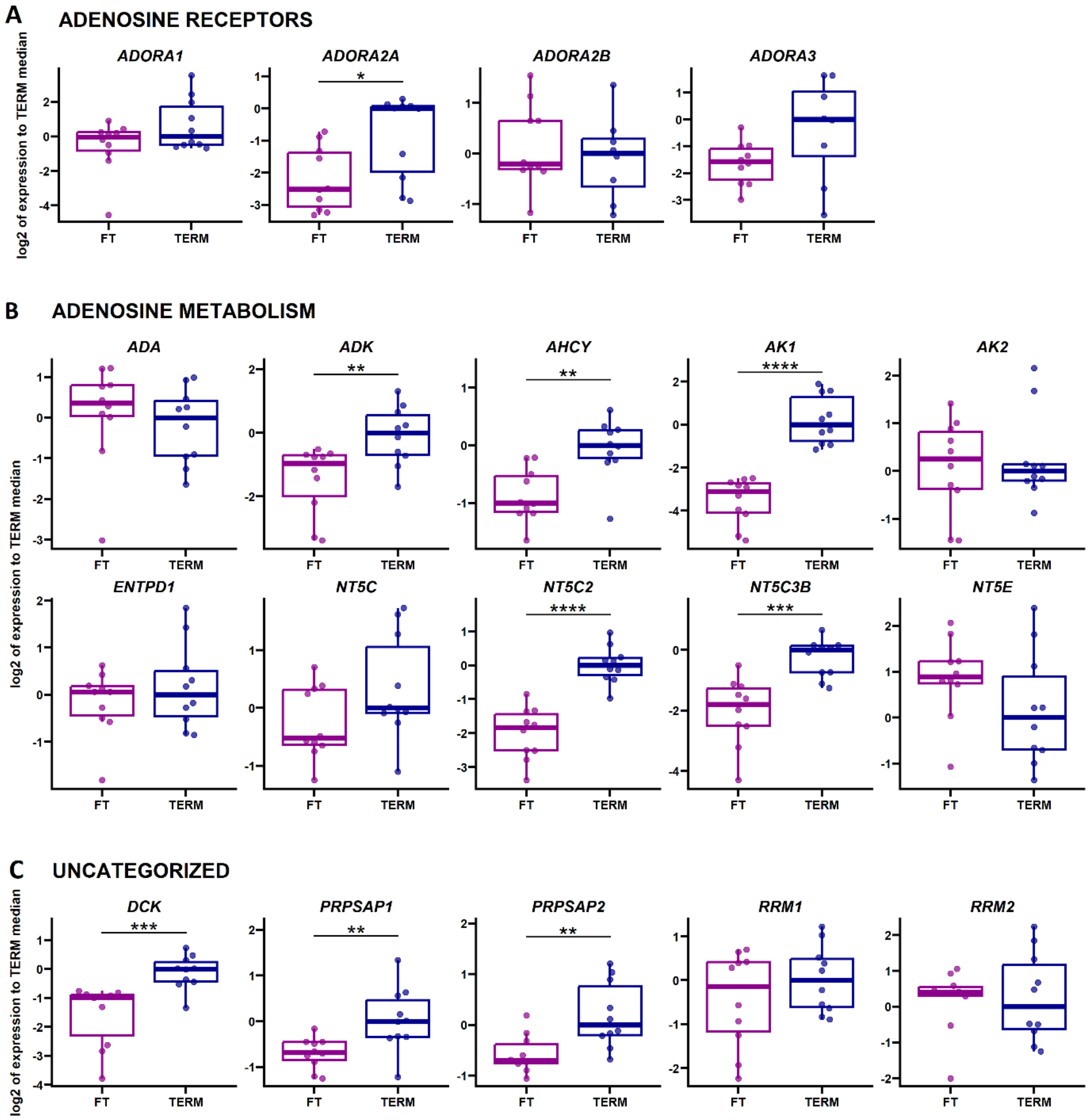

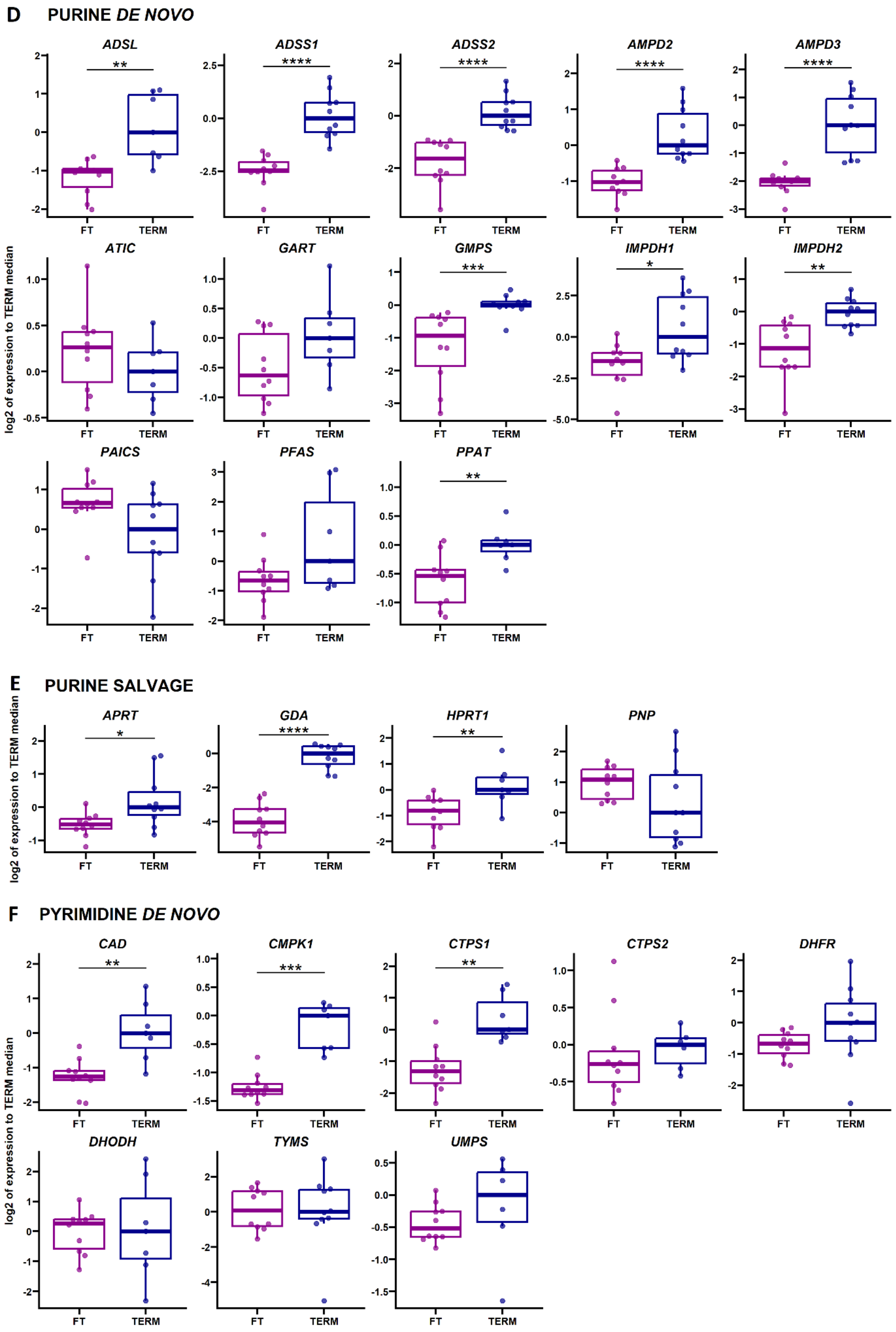

D PURINE DE NOVO
ADSL **
ADSS1 ****
ADSS2 ****
AMPD2 ****
AMPD3 ****
ATIC
GART
GMPS ***
IMPDH1 *
IMPDH2 **
PAICS
PFAS
PPAT **
log2 of expression to TERM median
FT
TERM
E PURINE SALVAGE
APRT *
GDA ****
HPRT1 **
PNP
log2 of expression to TERM median
F PYRIMIDINE DE NOVO
CAD **
CMPK1 ***
CTPS1 **
CTPS2
DHFR
DHODH
TYMS
UMPS
log2 of expression to TERM median

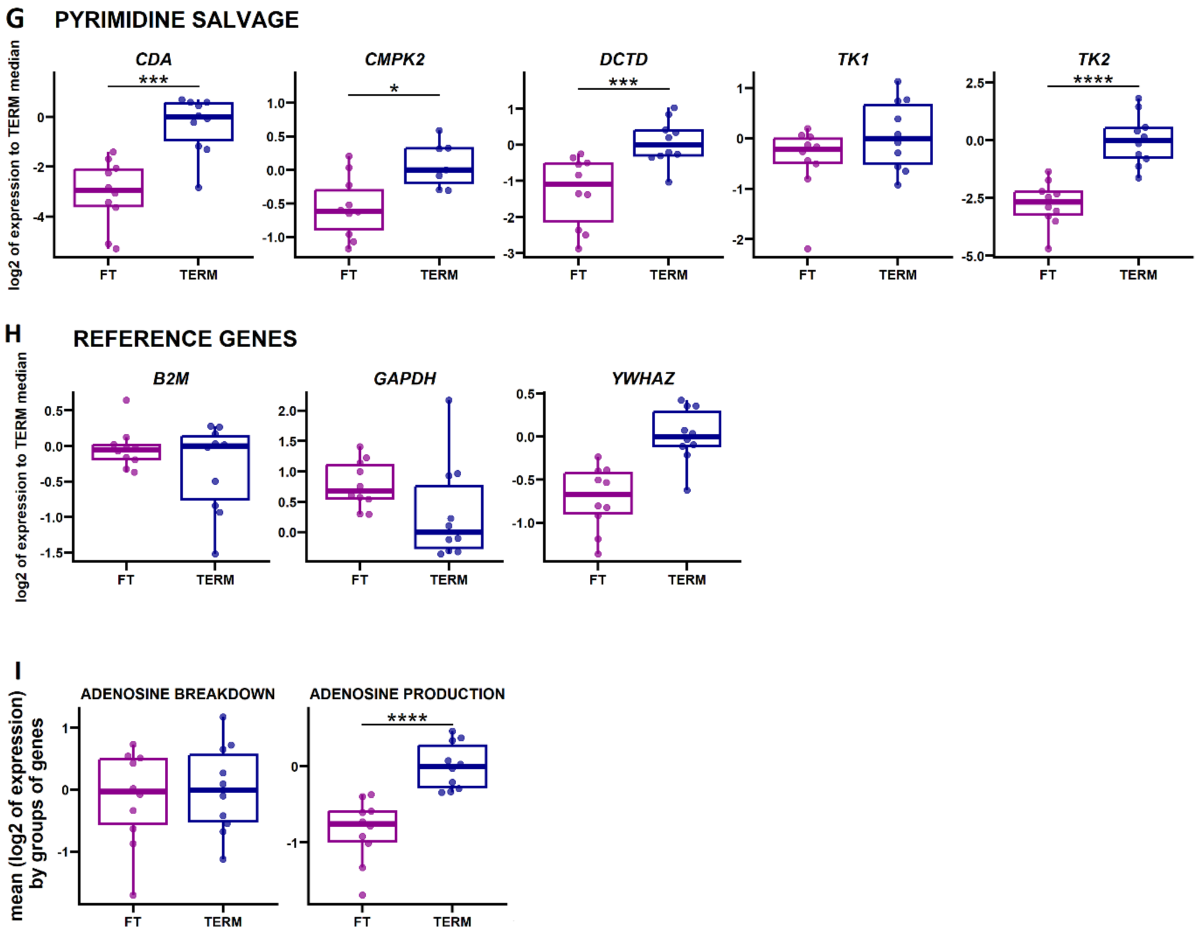

Supplementary Fig. 3. Comparison of the expressions centralized to the term mean expression for the first-trimester (FT) and term placenta across various gene categories**:** adenosine receptor (**A**), adenosine metabolism (**B**), group of uncategorized genes (**C**), purine *de novo* synthesis (**D**), purine salvage (**E**), pyrimidine *de novo* synthesis (**F**), pyrimidine salvage (**G**), reference genes (**H**), and comparison group on genes involved in the production and the breakdown of adenosine (**I**). Data are presented using box plots as log2 expressions of the mean of the term. Statistical analysis was performed using the non-parametric two-sample Wilcoxon (Mann-Whitney) test, (*$p < 0.05$; **$p < 0.01$; ***$p < 0.001$; ****$p < 0.0001$ relative to the term).

Comparison of gene expressions in preterm birth (PTB) placentas against term placentas

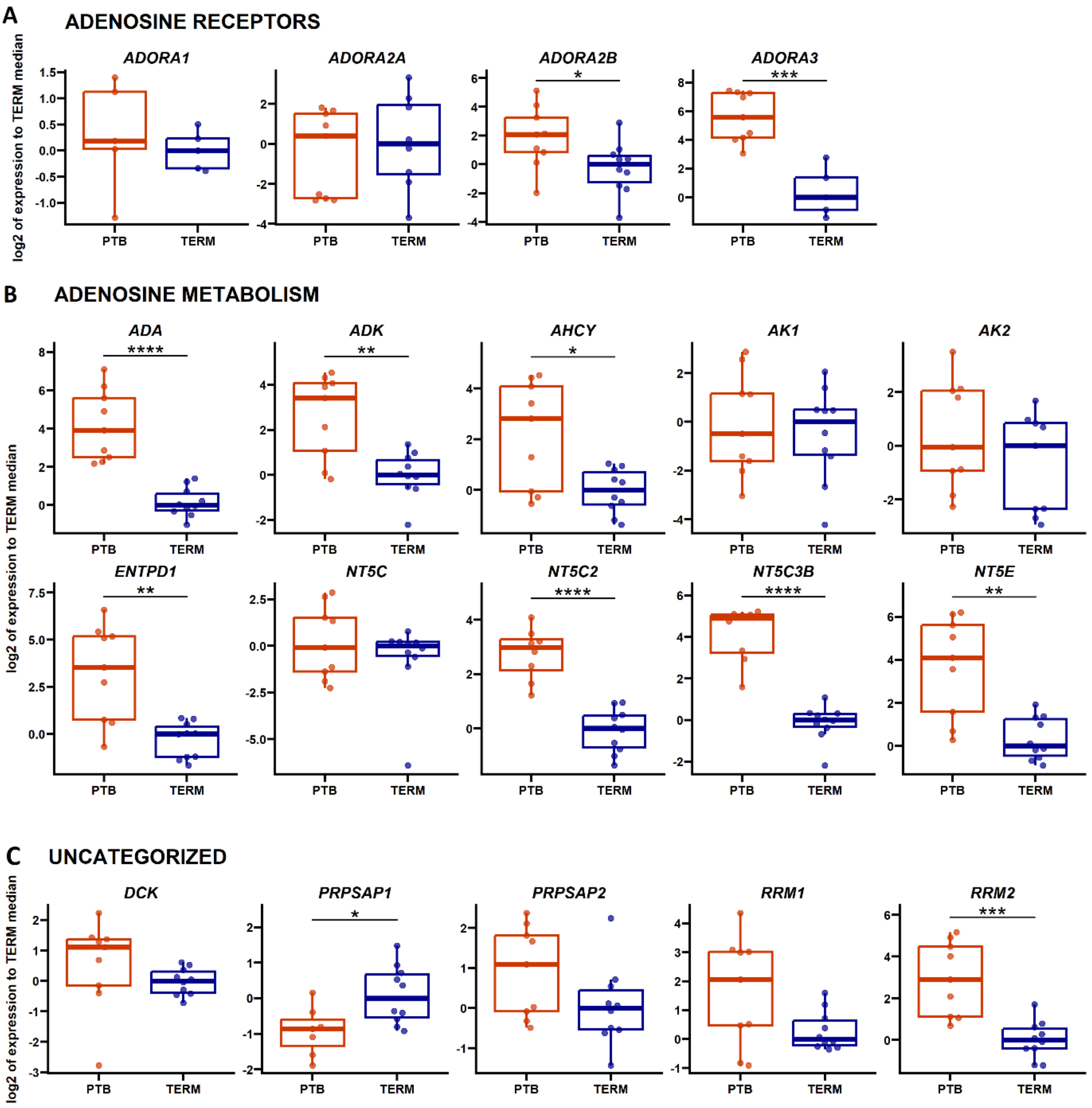

D PURINE *DE NOVO*

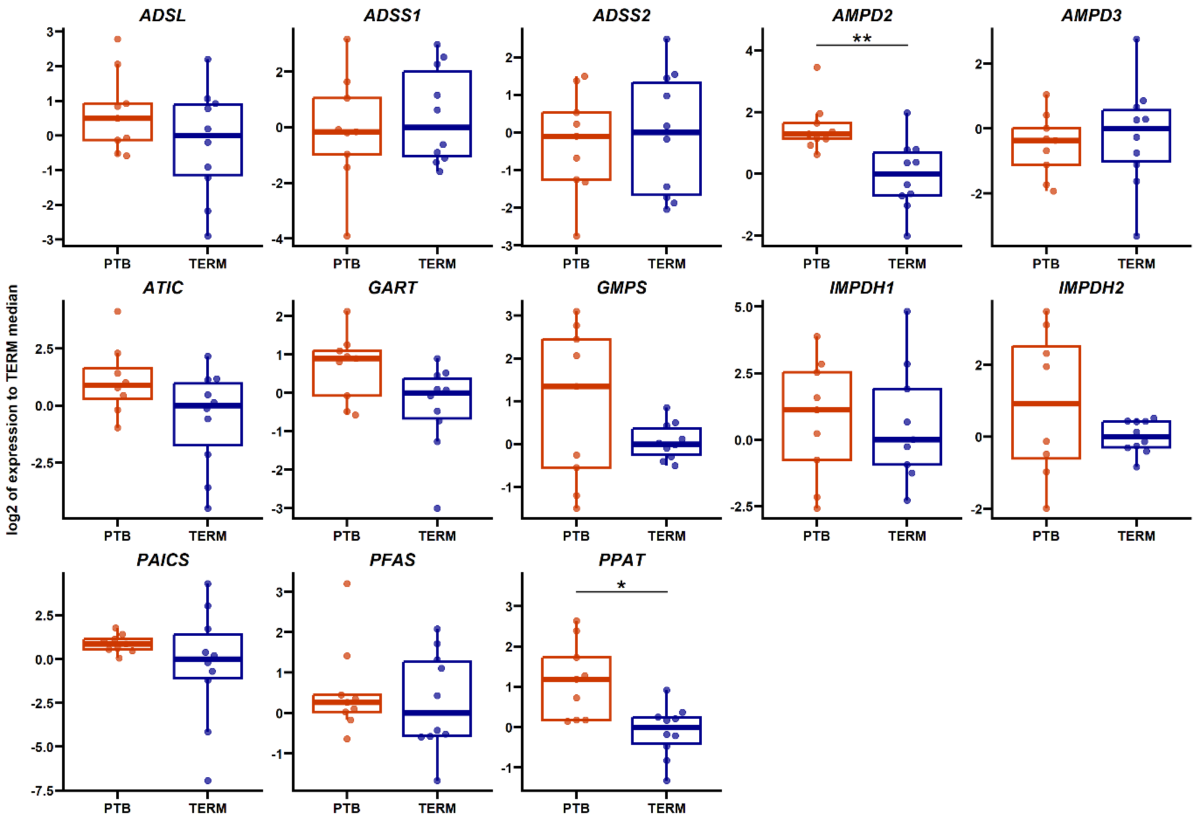

E PURINE SALVAGE

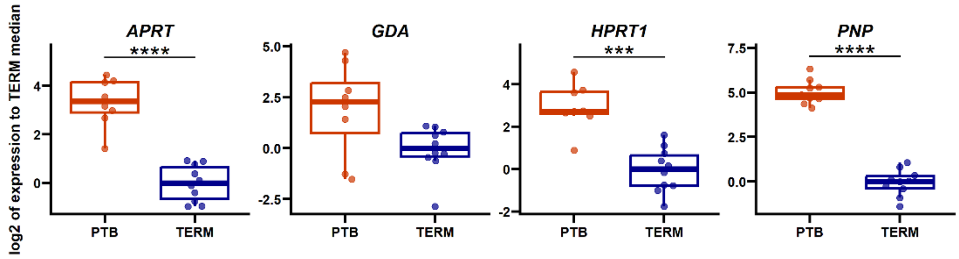

F PYRIMIDINE *DE NOVO*

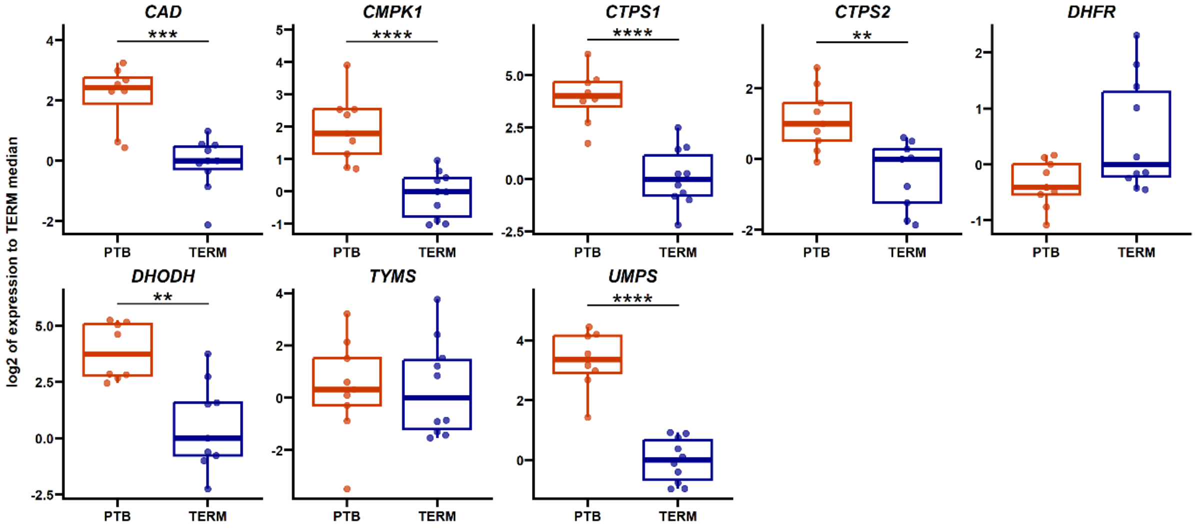

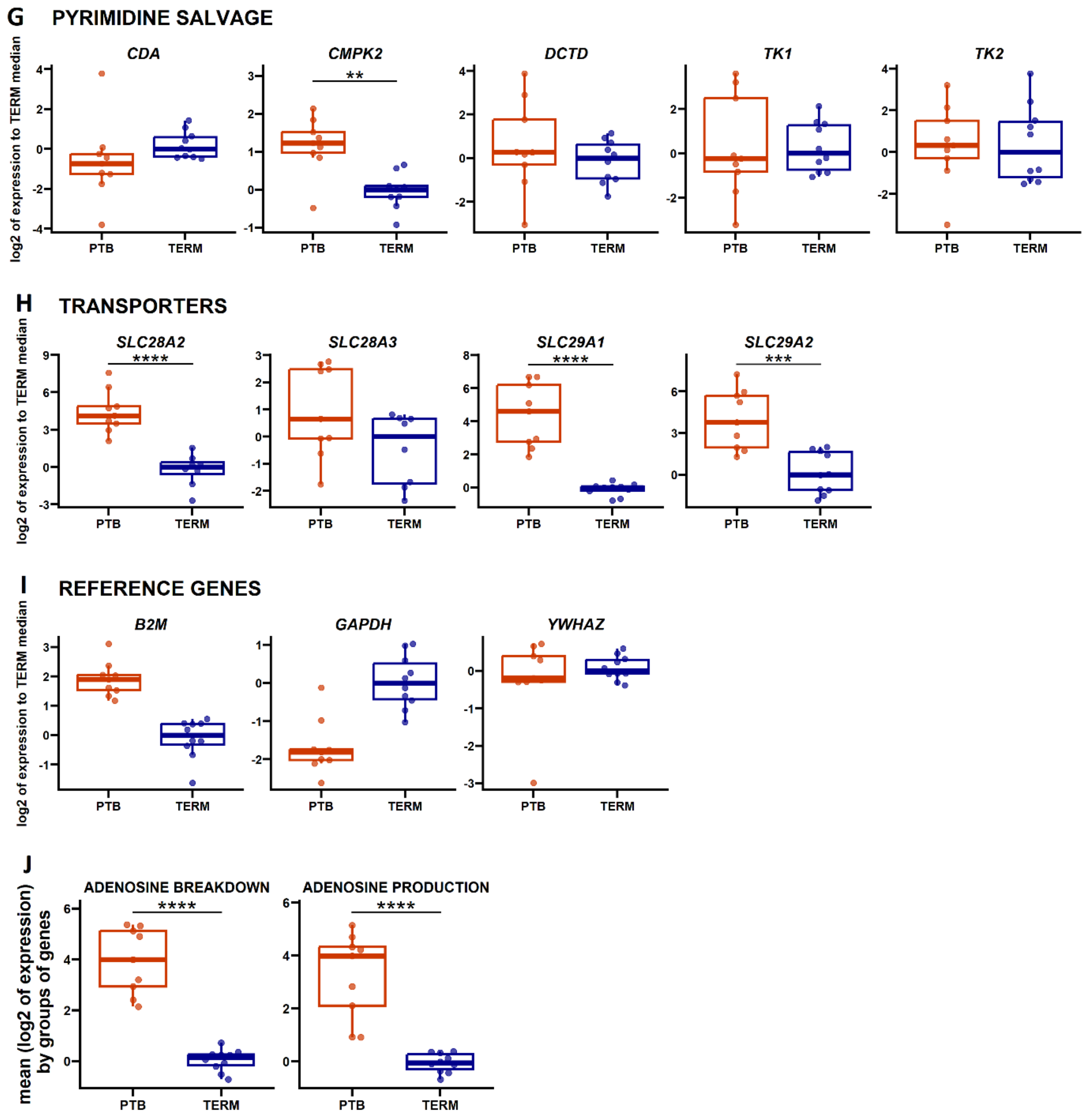

Supplementary Fig. 4. Comparison of expressions centralized to the term mean for preterm birth (PTB) and term placentas across various gene categories: adenosine receptor (**A**), adenosine metabolism (**B**), group of uncategorized genes (**C**), purine *de novo* synthesis (**D**), purine salvage (**E**), pyrimidine *de novo* synthesis (**F**), pyrimidine salvage (**G**), transporters (**H**), reference genes (**I**), and comparison group on genes involved in the production and the breakdown of adenosine (**J**). Data are using box plots presented as log2 expressions of the mean of the term. Statistical analysis was performed using the non-parametric two-sample Wilcoxon (Mann-Whitney) test, (*p < 0.05; **p < 0.01; ***p < 0.001; ****p < 0.0001 relative to the term).

Comparison of gene expressions in the rat placenta collected GD12, GD15, and GD20

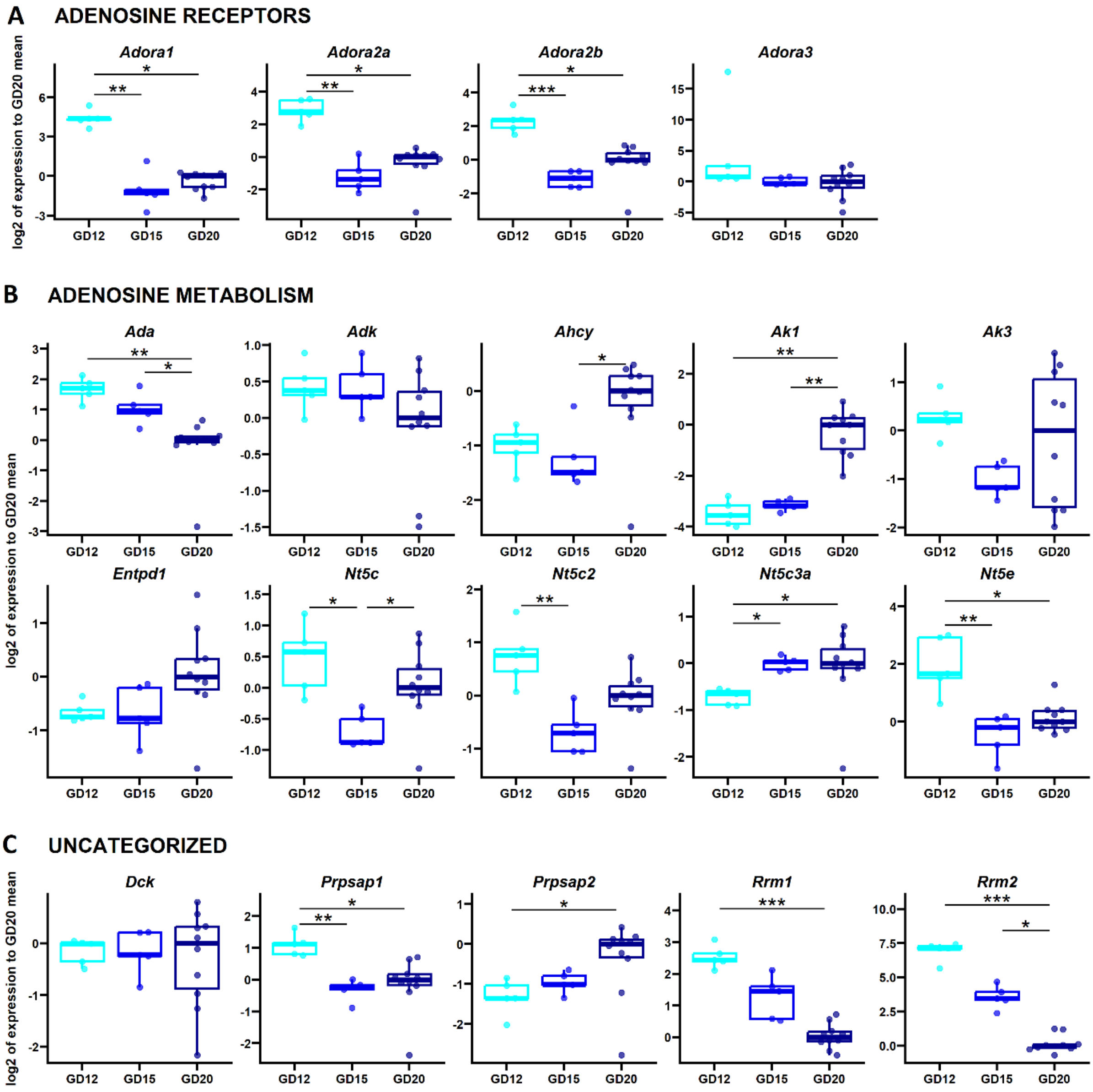

D PURINE *DE NOVO*

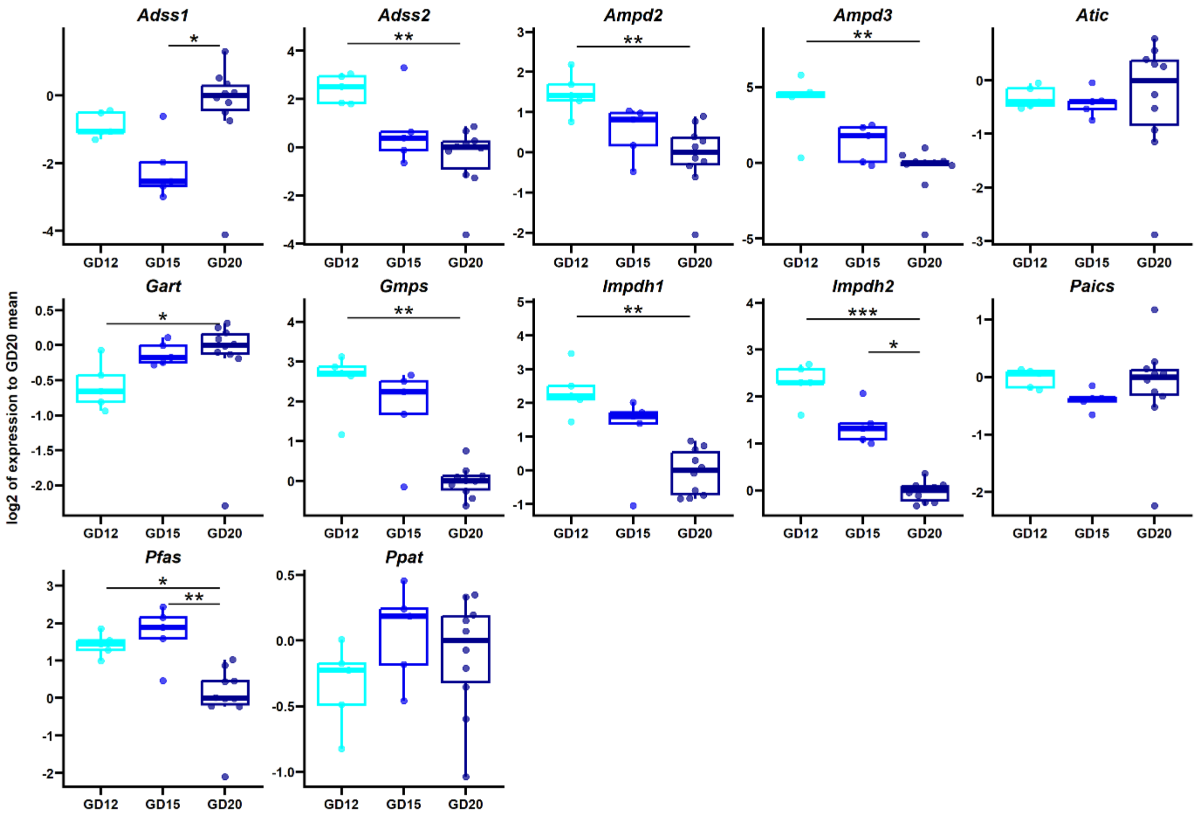

E PURINE SALVAGE

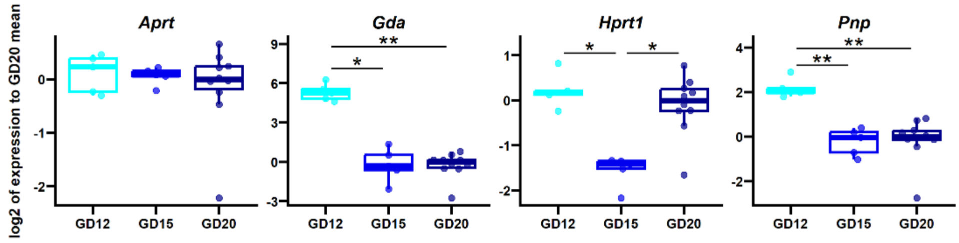

F PYRIMIDINE *DE NOVO*

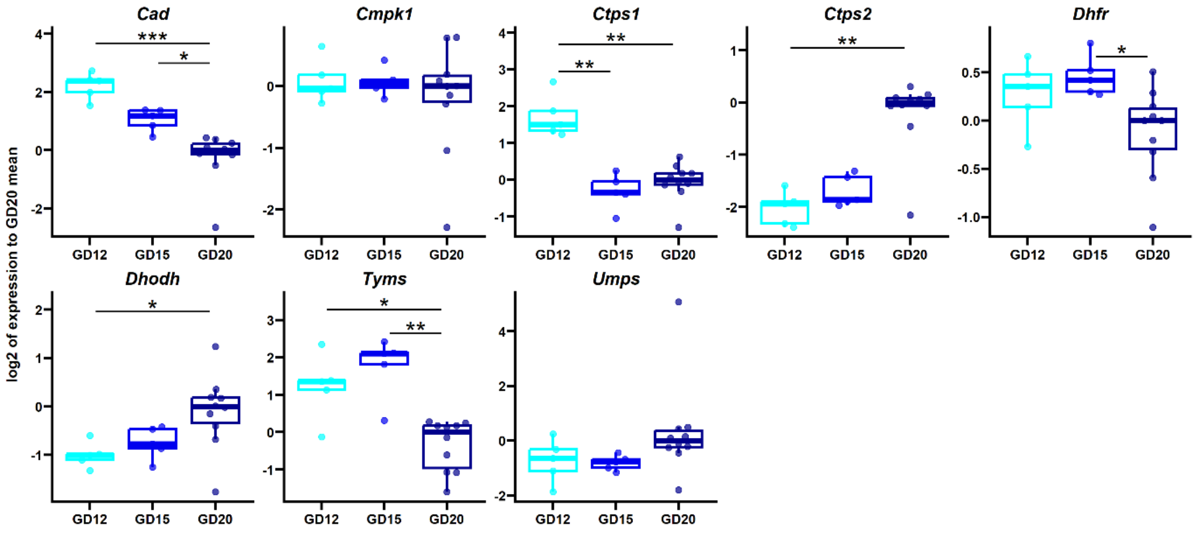

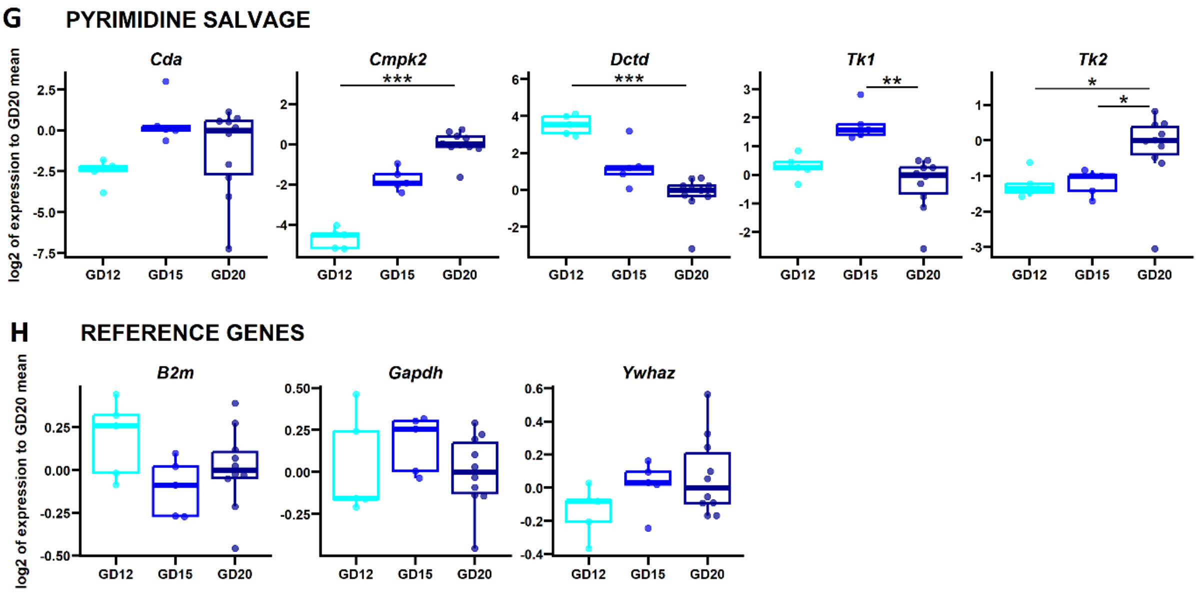

Supplementary Fig. 5. Comparison of the expressions centralized to the term mean expression for the placentas collected GD12, GD15, and GD20 for**:** adenosine receptors (**A**), adenosine metabolism (**B**), group of uncategorized genes (**C**), purine *de novo* synthesis (**D**), purine salvage (**E**), pyrimidine *de novo* synthesis (**F**), pyrimidine salvage (**G**), and reference genes (**H**). Data are presented using box plots as log2 expressions of the mean of the GD20. Statistical analysis was performed using the Dunn test (*p < 0.05; **p < 0.01; ***p < 0.001, relative to the GD20).

Information to the comparison of gene expressions in the rat placenta collected GD12, GD15, and GD20 - testing the sexual dimorphism in genes coding proteins involved in nucleos(t)ide placental handling

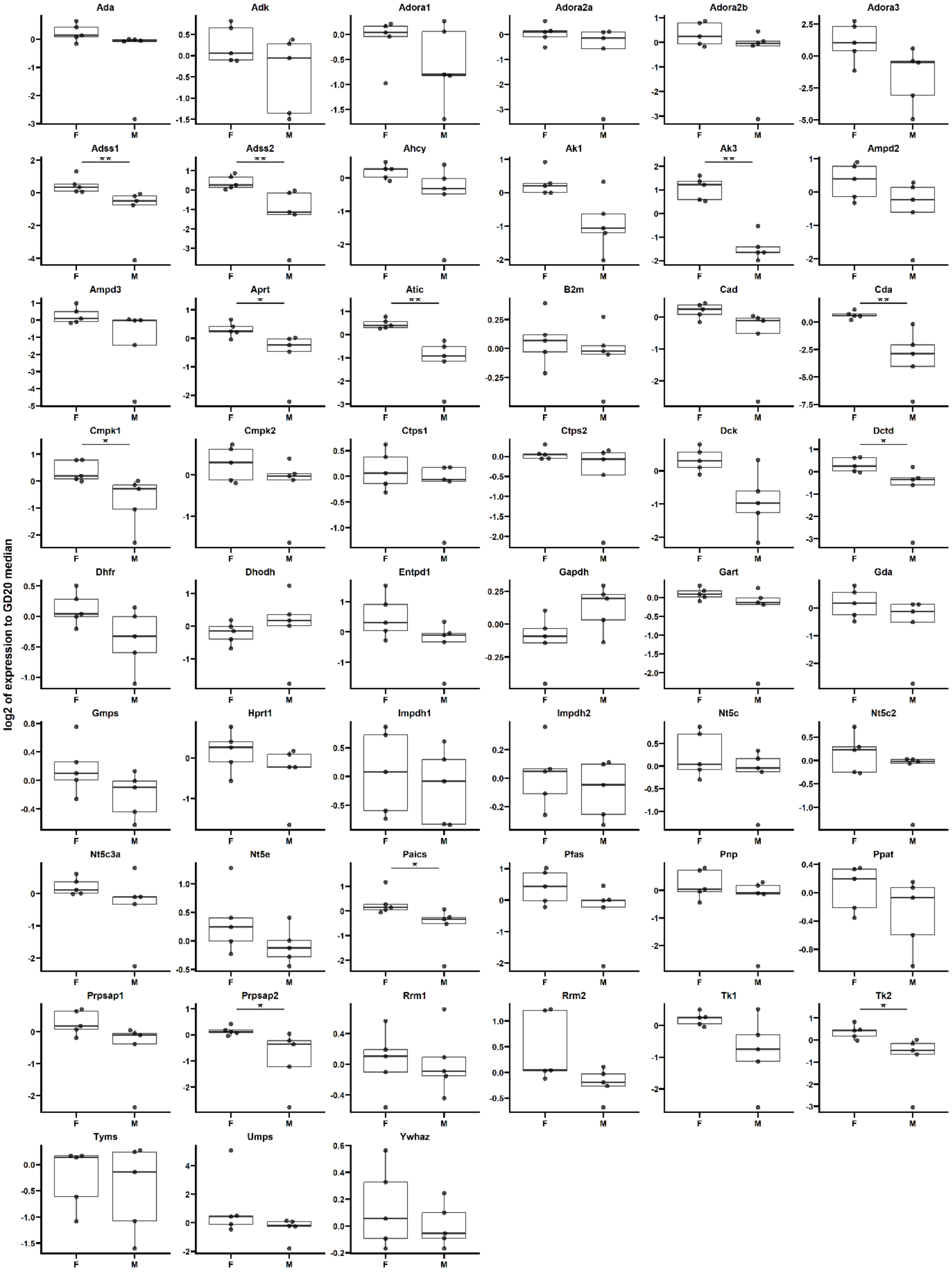

Supplementary Fig. 6. Comparison of expressions between male and female placentas collected at GD20. Data are presented using box plots as log2 expressions of the median of the GD20.

Statistical analysis was performed using the Wilcoxon (Mann-Whitney) test; (*p < 0.05; **p < 0.01).

Comparison of gene expressions in the human primary cytotrophoblast (CTB) and syncytiotrophoblast (STB)

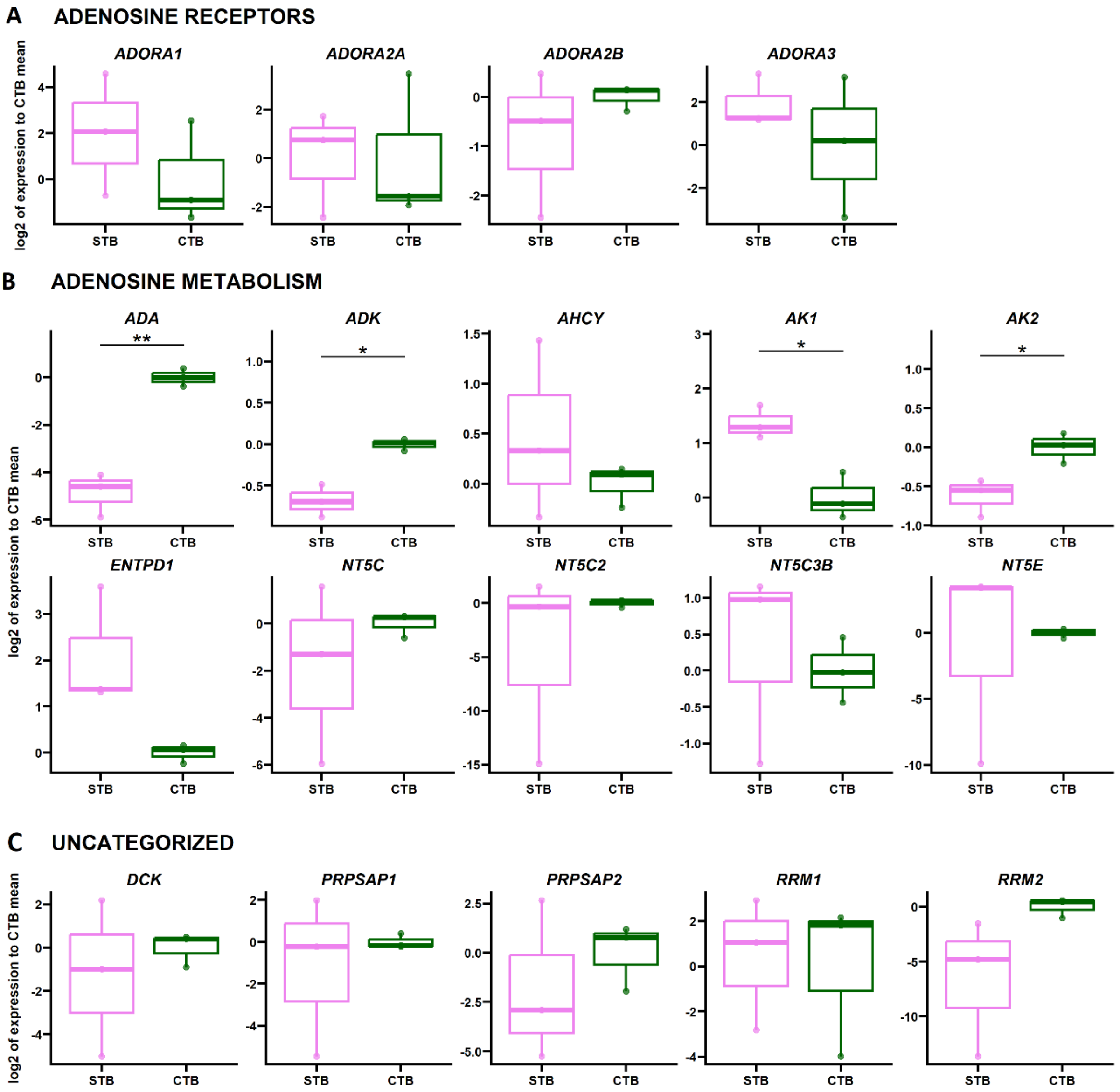

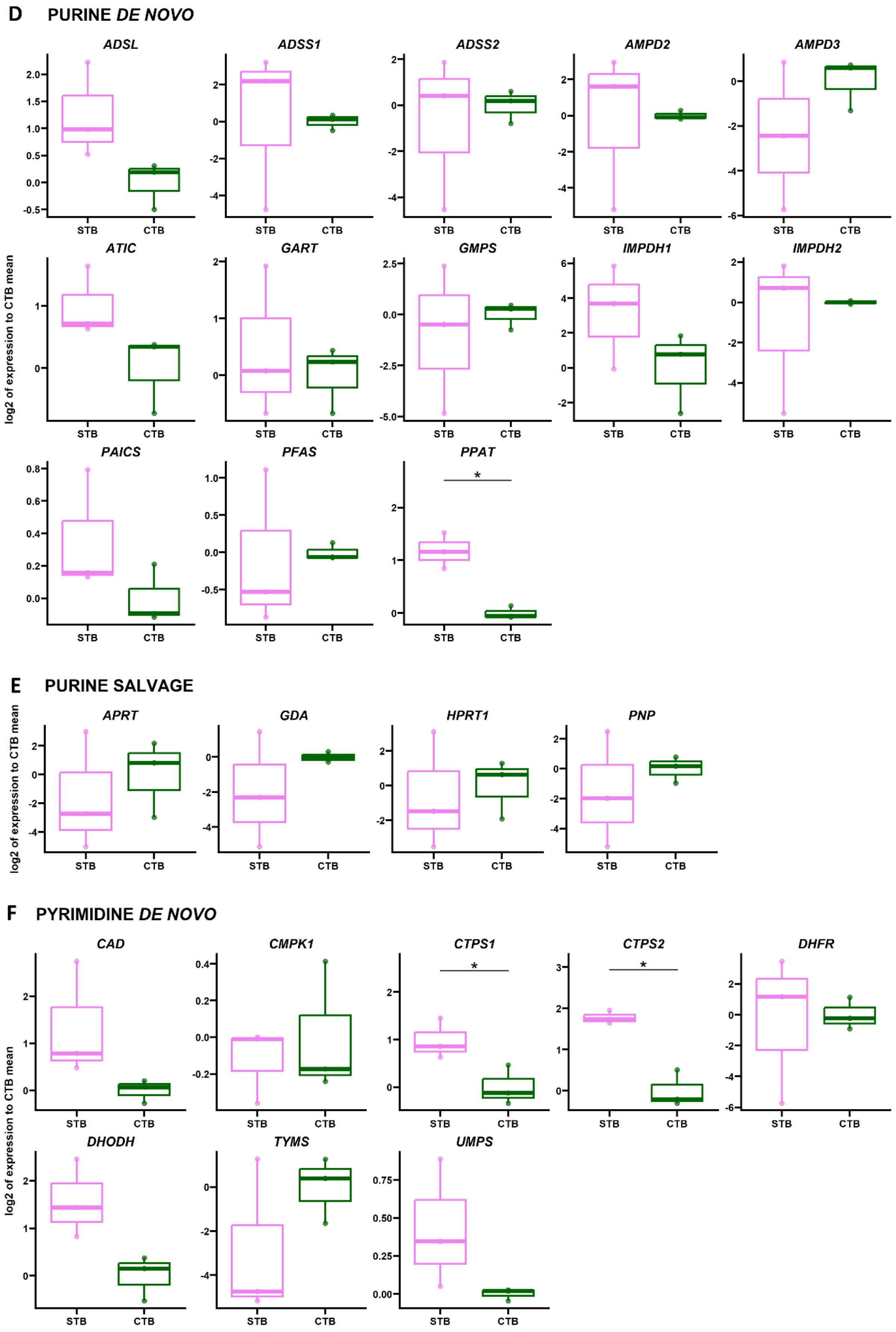
D PURINE DE NOVO
ADSL
ADSS1
ADSS2
AMPD2
AMPD3
ATIC
GART
GMPS
IMPDH1
IMPDH2
PAICS
PFAS
PPAT
*
E PURINE SALVAGE
APRT
GDA
HPRT1
PNP
F PYRIMIDINE DE NOVO
CAD
CMPK1
CTPS1
*
CTPS2
*
DHFR
DHODH
TYMS
UMPS
log2 of expression to CTB mean
STB
CTB

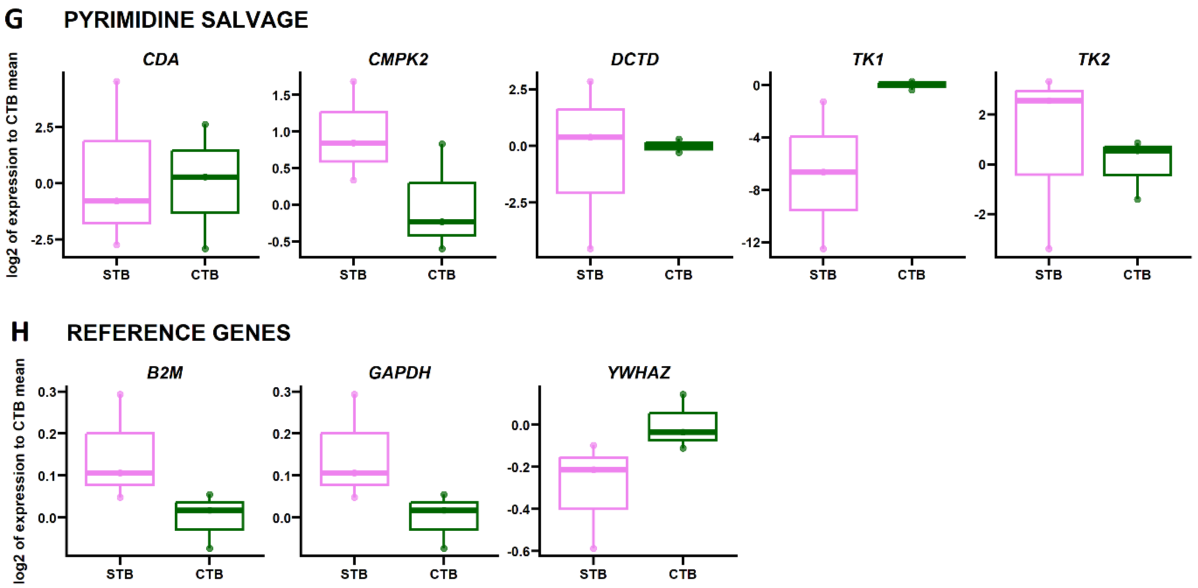

Supplementary Fig. 7. Comparison of expressions centralized to the syncytiotrophoblast (STB) mean expression for primary STB and cytotrophoblast (CTB) cells across various gene categories: adenosine receptors (**A**), adenosine metabolism (**B**), group of uncategorized genes (**C**), purine *de novo* synthesis (**D**), purine salvage (**E**), pyrimidine *de novo* synthesis (**F**), pyrimidine salvage (**G**), reference genes (**H**). *ADORA3* gene expression was not detected in BeWo cells. Data are presented using box plots as log2 expressions of the mean of the CTB. Statistical analysis was performed using the t-test (*p < 0.05; **p < 0.01, relative to the CTB).

Comparison of gene expressions in the BeWo cells and BeWo cells treated with forskolin.

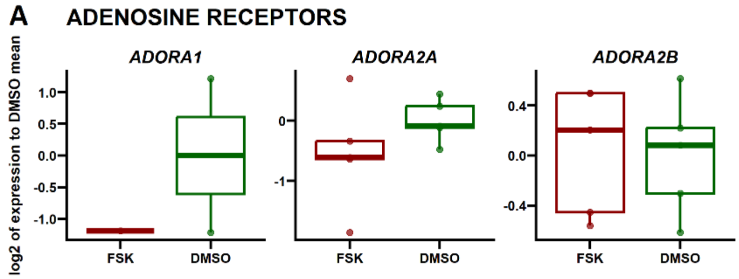

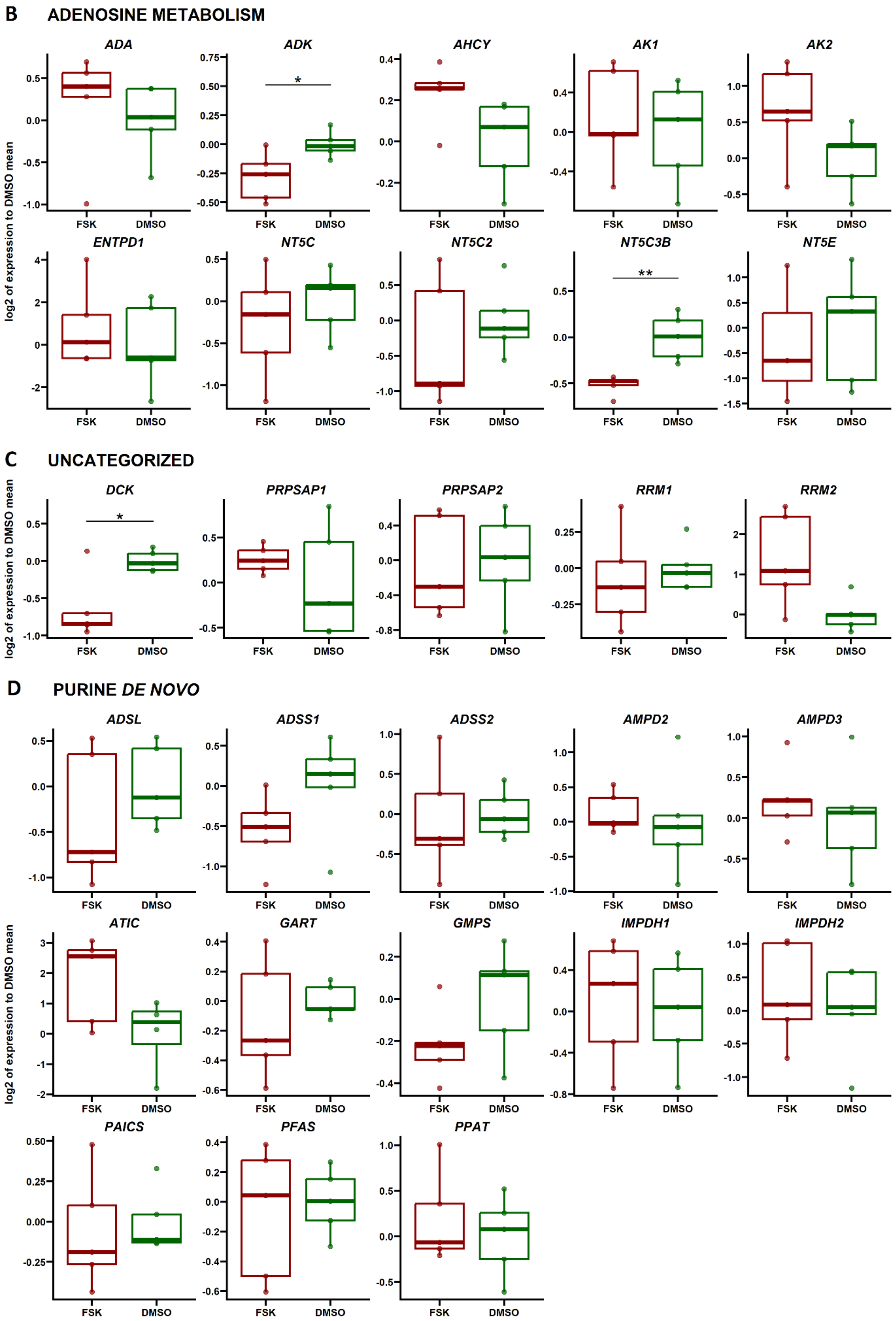
B ADENOSINE METABOLISM
ADA
ADK
*
AHCY
AK1
AK2
ENTPD1
NT5C
NT5C2
NT5C3B
**
NT5E
C UNCATEGORIZED
DCK
*
PRPSAP1
PRPSAP2
RRM1
RRM2
D PURINE DE NOVO
ADSL
ADSS1
ADSS2
AMPD2
AMPD3
ATIC
GART
GMPS
IMPDH1
IMPDH2
PAICS
PFAS
PPAT
log2 of expression to DMSO mean
FSK
DMSO

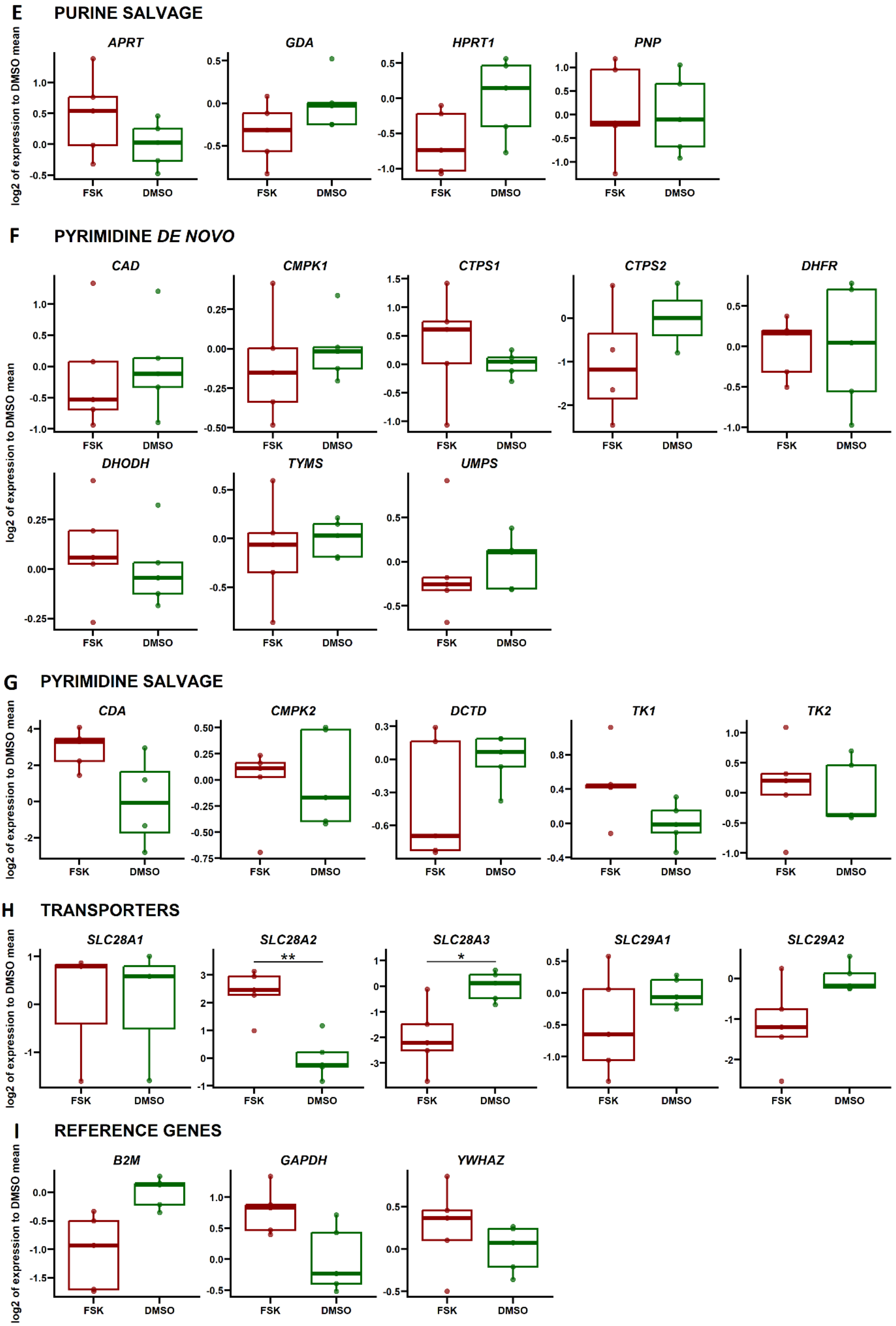

Supplementary Fig. 8. Comparison of expressions centralized to the mean expression of BeWo cells cultivated with DMSO for BeWo cells cultivated with DMSO (0.05%) and BeWo cells

treated with forskolin (FSK, 50 µM) across various gene categories: adenosine receptors (*ADORA3* gene expression was not detected) (**A**), adenosine metabolism (**B**), group of uncategorized genes (**C**), purine *de novo* synthesis (**D**), purine salvage (**E**), pyrimidine *de novo* synthesis (**F**), pyrimidine salvage (**G**), transporters (**H**), and reference genes (**G**). Data are presented using box plots as log2 expressions of the mean of the CTB. Statistical analysis was performed using the t-test (*p < 0.05; **p < 0.01, relative to the DMSO).

Expanded data on inosine, adenosine, adenosine monophosphate, and adenine levels in the term placenta and PTB placentas - premature rupture of membrane (PPROM) and preterm labor (PTL) samples discussed in discussion.

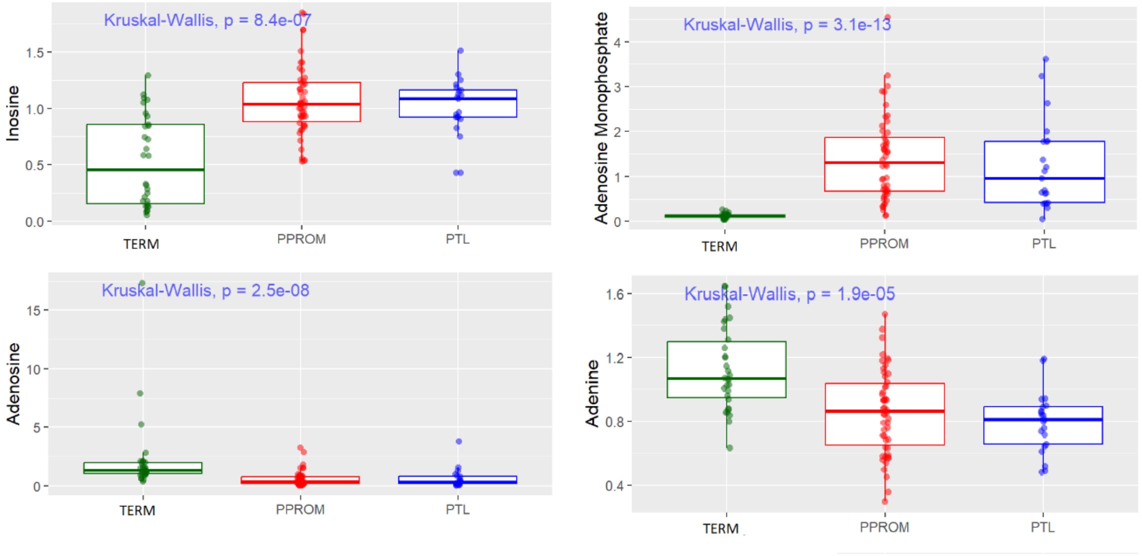

Supplementary Fig. 9. Inosine, adenosine, adenosine monophosphate, and adenine levels in term placentas and PTB placentas from donors with premature rupture of membranes (PPROM) and preterm labor (PTL). Data are presented as box plots of A_{sample}/A_{QC} [1]. Statistical analysis was performed using the non-parametric Kruskal-Wallis test ($p < 0.01$ was considered significant).

Supplementary Table 1. Detailed characteristics of preterm birth (PTB) and term pregnancies included in this study (expanded information for Methods section)

	PTB (n = 10)	Term (n =10)
**Age**	NA	33.6 ± 2.57
**Gestational age at delivery, week**	30.9 ± 3.51	38.4 ± 1.54
**Delivery weight, Kg**	68.60 ± 9.68	85 ± 14.93
**Newborn weight, Kg**	1.66 ± 0.59	3.34 ± 0.61
**Maternal BMI pre-pregnancy, Kg/m²**	22.4 ± 3.45	27 ± 6.21
**Maternal BMI at delivery, Kg/m²**	25.2 ± 3.14	31 ± 4.68
**Tissue weight, Kg**	0.24 ± 0.11	NA
**Diagnosis (PPROM), n**	5	NA
**Diagnosis (PTL), n**	5	NA
**Gender of the fetus, n**	NA	5 males and 5 females
**Maternal serum CRP concentration at delivery (mg/l)**	10.73 ± 10,82	NA
**Maternal serum WBC count at delivery (×109 L)**	16.93 ± 6.93	NA
**Amniotic fluid IL-6 conc. at admission (ng/ml)**	10556,86 ± 14438.38	NA
**Corticosteroids**	8 out of 10	1 out of 10
**Antibiotics**	9 out of 10	NA
**Tocolytics**	3 out of 10	NA
**Thyroid hormone**	NA	2 out of 10
**Iron supplements**	NA	1 out of 10
**Anticoagulant drugs**	NA	1 out of 10
**Bronchodilators**	NA	1 out of 10
**Antihypertensive drugs**	NA	1 out of 10

Results are expressed as the mean ± SD; *n* = 10; PPROM, preterm premature rupture of membranes; PTL, preterm labor; CRP, C-reactive protein; WBC, white blood cells

Supplementary Table 2. Detailed characteristics of first-trimester (FT) and term pregnancies included in this study (expanded information for Methods section)

		FT (n = 10)	Term (n =10)
**Age**		27.9 ± 7.66	36.5 ± 2.97
**Gestational age at delivery, week**		9.7 ± 1.17	39.4 ± 0.54
**Newborn weight, Kg**		NA	3.44 ± 0.37
**Maternal BMI in prepregancy, Kg/m²**		NA	23 ± 2.77
**Maternal BMI delivery, Kg/m²**		NA	28 ± 3.07
**Tissue weight, Kg**		NA	NA
**Smoking cigarettes**		5 out of 10	NA

Supplementary Table 2 (continuation). Detailed characteristics of first-trimester (FT) and term pregnancies included in this study (expanded information for Methods section)

**Anxiolytic drugs**		**2 out of 10**	**5 out of 10**
**Thyroid hormones**		NA	1 out of 10
**Corticosteroids**		NA	NA

Results are expressed as the mean ± SD; *n* = 10.

Supplementary Table 3. Descriptive statistics of pregnant Wistar rats used in this study (expanded information for Methods section)

<table>
<tr><th>Parameter</th><th>GD12</th><th>GD15</th><th>GD20</th></tr>
<tr><td>Number of fetuses (n)</td><td>11.2 ± 5.41</td><td>13.6 ± 1.85</td><td>16.2 ± 0.748</td></tr>
<tr><td>Rat weight (Kg)</td><td>0.297 ± 0.010</td><td>0.289 ± 0.042</td><td>0.40 ± 0.020</td></tr>
<tr><td>Placental weight (Kg)</td><td rowspan="2">0.000081 ± 0.000024 [1]</td><td>0.00016 ± 0.000018</td><td>0.000445 ± 0.00006</td></tr>
<tr><td>Fetus weight (Kg)</td><td>NA</td><td>0.002 ± 0.000058</td></tr>
</table>

[1] Concepti (consisting of embryos and extra-embryonic tissues). Results are expressed as the mean ± SD; n = 5.

Supplementary Table 4. Human expression assays used for quantifying human gene levels (expanded information for Methods section)

	Assay code	Gene name	Gene symbol
ADORA	Hs00181231_m1	adenosine A1 receptor	*ADORA1*
	Hs00169123_m1	adenosine A2A receptor	*ADORA2A*
	Hs00386497_m1	adenosine A2b receptor	*ADORA2B*
	Hs00181232_m1	adenosine A3 receptor	*ADORA3*
ADENOSINE METABOLISM	Hs00274359_m1	5', 3'-nucleotidase, cytosolic	*NT5C*
	Hs01056741_m1	5'-nucleotidase, cytosolic II	*NT5C2*
	Hs00369454_m1	5'-nucleotidase, cytosolic IIIB	*NT5C3b*
	Hs00417073_m1	adenosine kinase	*ADK*
	Hs01110945_m1	adenosine deaminase	*ADA*
	Hs00898137_g1	adenosylhomocysteinase	*AHCY*
	Hs04234686_m1	5'-nucleotidase ecto	*NT5E (CD-73)*
	Hs00969559_m1	ectonucleoside triphosphate diphosphohydrolase 1	*ENTPD1 (CD-39)*
	Hs00176119_m1	adenylate kinase 1	*AK1*
	Hs01650722_m1	adenylate kinase 2	*AK2*
UNCATEGORIZED	Hs01048899_m1	phosphoribosyl pyrophosphate synthetase associated protein 1	*PRPSAP1*
	Hs00160482_m1	phosphoribosyl pyrophosphate synthetase associated protein 2	*PRPSAP2*
	Hs01040726_m1	deoxycytidine kinase	*DCK*
	Hs01040698_m1	ribonucleotide reductase catalytic subunit M1	*RRM1*
	Hs00357247_g1	ribonucleotide reductase regulatory subunit M2	*RRM2*
PURINE *DE NOVO*	Hs01075807_m1	adenylosuccinate lyase	*ADSL*
	Hs00765651_m1	adenylosuccinate synthase	*ADSS2*
	Hs00411846_m1	adenylosuccinate synthase 1	*ADSS1*
	Hs00187821_m1	adenosine monophosphate deaminase 2	*AMPD2*
	Hs00983048_m1	adenosine monophosphate deaminase 3	*AMPD3*
	Hs00269671_m1	5-aminoimidazole-4-carboxamide ribonucleotide formyltransferase/IMP cyclohydrolase	*ATIC*
	Hs00894582_m1	phosphoribosylglycinamide formyltransferase,phosphoribosylglycinamide synthetase, phosphoribosylaminoimidazole synthetase	*GART*
	Hs00269500_m1	guanine monophosphate synthase	*GMPS*
	Hs04190080_gH	Inosine monophosphate dehydrogenase 1	*IMPDH1*

Supplementary Table 4 (continuation). Human expression assays used for quantifying human gene levels (expanded information for Methods section)

	Assay code	Gene name	Gene symbol
PURINE *DE NOVO*	Hs00168418_m1	inosine monophosphate dehydrogenase 2	*IMPDH2*
	Hs00601264_m1	phosphoribosyl pyrophosphate amidotransferase	*PPAT*
	Hs00389822_m1	phosphoribosylformylglycinamidine synthase	*PFAS*
	Hs00935017_gH	phosphoribosylaminoimidazole carboxylase and phosphoribosylaminoimidazolesuccinocarboxamide synthase	*PAICS*
PURINE SALVAGE	Hs00356991_m1	adenine phosphoribosyltransferase	*APRT*
	Hs00393722_m1	guanine deaminase	*GDA*
	Hs00165367_m1	purine nucleoside phosphorylase	*PNP*
	Hs02800695_m1	hypoxanthine phosphoribosyltransferase 1	*HPRT1*
PYRIMIDINE *DE NOVO*	Hs00983188_m1	carbamoyl-phosphate synthetase 2, aspartate transcarbamylase, and dihydroorotase	*CAD*
	Hs01074420_g1	cytidine/uridine monophosphate kinase 1	*CMPK1*
	Hs01041851_m1	cytidine triphosphate synthase 1	*CTPS1*
	Hs00219845_m1	cytidine triphosphate synthase 2	*CTPS2*
	Hs00758822_s1	dihydrofolate reductase	*DHFR*
	Hs00361406_m1	dihydroorotate dehydrogenase	*DHODH*
	Hs00426586_m1	thymidylate synthetase	*TYMS*
	Hs00923517_m1	uridine monophosphate synthetase	*UMPS*
PYRIMIDINE SALVAGE	Hs00156401_m1	cytidine deaminase	*CDA*
	Hs01013364_m1	cytidine/uridine monophosphate kinase 2	*CMPK2*
	Hs01126095_m1	dCMP deaminase	*DCTD*
	Hs01062125_m1	thymidine kinase 1	*TK1*
	Hs00936914_m1	thymidine kinase 2	*TK2*
TRANSPORTERS	Hs00984403_m1	solute carrier family 28-member 1	*SLC28A1 (CNT1)*
	Hs01035846_m1	solute carrier family 28-member 2	*SLC28A2 (CNT2)*
	Hs00910439_m1	solute carrier family 28-member 3	*SLC28A3 (CNT3)*

Supplementary Table 4 (continuation). Human expression assays used for quantifying human gene levels (expanded information for Methods section)

	Assay code	Gene name	Gene symbol
TRANSPORTERS	Hs01085704_g1	solute carrier family 29-member 1	*SLC29A1 (ENT1)*
	Hs00155426_m1	solute carrier family 29-member 2	*SLC29A2 (ENT2)*
REFERENCE	Hs02758991_g1	glyceraldehyde-3-phosphate dehydrogenase	*GAPDH*
	Hs00187842_m1	beta-2-microglobulin	*B2M*
	Hs01122445_g1	tyrosine 3-monooxygenase/tryptophan 5-monooxygenase activation protein zeta	*YHWAZ*
FUSION MARKER	Hs00205893_m1	endogenous retrovirus group W member 1, envelope	*ERVW-1*

Supplementary Table 5. Rat expression assays used for quantifying rat gene levels (expanded information for Methods section)

	Assay code	Gene name	Gene symbol
ADORA	Rn00567668_m1	adenosine A1 receptor	*Adora1*
	Rn00583935_m1	adenosine A2A receptor	*Adora2a*
	Rn00567697_m1	adenosine A2B receptor	*Adora2b*
	Rn00563680_m1	adenosine A3 receptor	*Adora3*
ADENOSINE METABOLISM	Rn01464713_g1	5', 3'-nucleotidase, cytosolic	*Nt5c*
	Rn01444683_g1	5'-nucleotidase, cytosolic II	*Nt5c2*
	Rn01451791_m1	5'-nucleotidase, cytosolic IIIA	*Nt5c3a*
	Rn01537044_m1	adenosine kinase	*Adk*
	Rn00590253_m1	adenosine deaminase	*Ada*
	Rn00820837_g1	adenosylhomocysteinase	*Ahcy*
	Rn00665212_m1	5' nucleotidase, ecto	*Nt5e*
	Rn00574887_m1	ectonucleoside triphosphate diphosphohydrolase 1	*Entpd1 (Cd-39)*
	Rn00577377_m1	adenylate kinase 1	*Ak1*
	Rn00566296_m1	adenylate kinase 3	*Ak3*
UNCATEGORIZED	Rn00574822_m1	phosphoribosyl pyrophosphate synthetase associated protein 1	*Prsap1*
	Rn00589168_m1	phosphoribosyl pyrophosphate synthetase associated protein 2	*Prpsap2*
	Rn00577340_m1	deoxycytidine kinase	*Dck*
	Rn01510539_m1	ribonucleotide reductase catalytic subunit M1	*Rrm1*
	Rn01768870_g1	ribonucleotide reductase regulatory subunit M2	*Rrm2*
PURINE *DE NOVO*	Rn02103847_s1	adenylosuccinate synthase	*Adss2*
	Rn01430183_m1	adenylosuccinate synthase 1	*Adss1*
	Rn01522390_m1	adenosine monophosphate deaminase 2	*Ampd2*
	Rn00580635_m1	adenosine monophosphate deaminase 3	*Ampd3*
	Rn00578818_m1	5-aminoimidazole-4-carboxamide ribonucleotide formyltransferase/IMP cyclohydrolase	*Atic*
	Rn01477298_m1	phosphoribosylglycinamide formyltransferase, phosphoribosylglycinamide synthetase, phosphoribosylaminoimidazole synthetase	*Gart*
	Rn01495015_m1	guanine monophosphate synthase	*Gmps*
	Rn01455844_m1	inosine monophosphate dehydrogenase 1	*Impdh1*
	Rn01640111_g1	inosine monophosphate dehydrogenase 2	*Impdh2*
	Rn01413080_m1	phosphoribosyl pyrophosphate amidotransferase	*Ppat*
	Rn01464102_m1	phosphoribosylformylglycinamidine synthase	*Pfas*

Supplementary Table 5 (continuation). Rat expression assays used for quantifying rat gene levels (expanded information for Methods section)

	Assay code	Gene name	Gene symbol
PURINE *DE NOVO*	Rn01413652_g1	phosphoribosylaminoimidazole carboxylase and phosphoribosylaminoimidazolesuccinocarboxamide synthase	*Paics*
PURINE SALVAGE	Rn01432775_m1	adenine phosphoribosyl transferase	*Aprt*
	Rn00582297_m1	guanine deaminase	*Gda*
	Rn01414648_g1	purine nucleoside phosphorylase	*Pnp*
	Rn01527840_m1	hypoxanthine phosphoribosyltransferase 1	*Hprt1*
PYRIMIDINE *DE NOVO*	Rn01472905_m1	carbamoyl-phosphate synthetase 2, aspartate transcarbamylase, and dihydroorotase	*Cad*
	Rn01761436_m1	cytidine/uridine monophosphate kinase 1	*Cmpk1*
	Rn00516751_m1	CTP synthase 1	*Ctps1*
	Rn01416312_m1	CTP synthase 2	*Ctps2*
	Rn04342282_g1	dihydrofolate reductase	*Dhfr*
	Rn01432611_m1	dihydroorotate dehydrogenase	*Dhodh*
	Rn01418709_m1	thymidylate synthetase	*Tyms*
	Rn01475102_m1	uridine monophosphate synthetase	*Umps*
PYRIMIDINE SALVAGE	Rn01519153_m1	cytidine deaminase	*Cda*
	Rn01473059_m1	cytidine/uridine monophosphate kinase 2	*Cmpk2*
	Rn01488492_m1	dCMP deaminase	*Dctd*
	Rn01456339_g1	thymidine kinase 1	*Tk1*
	Rn01536809_m1	thymidine kinase 2	*Tk2*
REFERENCE	Rn01462662_g1	glyceraldehyde-3-phosphate dehydrogenase	*Gapdh*
	Rn00560865_m1	beta-2 microglobulin	*B2m*
	Rn00755072_m1	tyrosine 3-monooxygenase/tryptophan 5-monooxygenase activation protein, zeta	*Yhwaz*

Supplementary Table 6. Summary of potential gene/protein roles in pathology and placental function

Gene/Protein	Upregulated in PTB	Upregulated in pathology	Type(s) of pathology	Possible function in pathology	Potential protective role in placenta	Reference
**ADORA2B**	Yes	Yes	Preeclampsia	-	-	Ref.[2]
**ADORA3**	Yes	Yes	Preeclampsia	-	-	Ref.[3]
**NT5E**	Yes	Yes	Preeclampsia	proliferation of trophoblast	Placental development	Ref.[4]
**ADA**	Yes	Yes	Preeclampsia	Cellular immunity	Immunoregulation	Ref. [5]
**AHCY**	Yes	Yes	Preeclampsia	DNA methylation	Placental epigenetic modification	Ref.[6,7]
**AMPD2**	Yes	-	-	Anti-inflammatory effect mediated by IMP	Immunoregulation	Ref.[8]
**PPAT**	Yes	Yes	Thyroid cancer	Cancer growth	Placental development	Ref.[9]
**APRT**	Yes	-	-	Energy homeostasis	Placental energy homeostasis	Ref.[10]
**HPRT**	Yes	Yes	Prostate cancer	Regulation of the immune system by producing adenosine and guanosine	Immunoregulation	Ref.[11]
**PNP**	Yes	Yes	Breast and colorectal cancer	Cancer growth	Placental development	Ref.[12]
**CMPK1**	Yes	Yes	Triple-negative breast cancer	Cancer growth	Placental development	Ref.[13]
**CMPK2**	Yes	Yes	Cervical cancer	Cancer Growth	Placental development	Ref.[14]
**CAD**	Yes	Yes	Breast cancer	Cancer growth and metastasis	Placental development	Ref [15]

Supplementary Table 6 (continuation). Summary of potential gene/protein roles in pathology and placental function

Gene/Protein	Upregulated in PTB	Upregulated in pathology	Type(s) of Pathology	Possible function in Pathology	Potential Protective Role in Placenta	Reference
**CTPS1**	Yes	Yes	Myeloma cancer	Cancer growth	Placental development	Ref.[16]
**CTPS2**	Yes	Yes	Chronic lymphocytic leukemia	Genomic stability and repair mechanism	placental DNA integrity and repair processes	Ref.[17]
**DHODH**	Yes	**-**	**-**	Cancer growth	Placental development	Ref.[18]
**UMPS**	Yes	Yes	Malignant pleural mesothelioma	Cancer growth	Placental development	Ref.[19]
**RRM2**	Yes	Yes	Prostate cancer	DNA repair and synthesis	Placental DNA repair processes	Ref.[20]
**PRPSAP1**	Yes	Yes	Neuroblastoma	Cancer growth	Placental growth	Ref.[21]

Reference

1 Cifkova, E. *et al.* Metabolomic analysis of the human placenta reveals perturbations in amino acids, purine metabolites, and small organic acids in spontaneous preterm birth. *Excli j* **23**, 264-282, doi:10.17179/excli2023-6785 (2024).

2 Iriyama, T. *et al.* Elevated placental adenosine signaling contributes to the pathogenesis of preeclampsia. *Circulation* **131**, 730-741, doi:10.1161/circulationaha.114.013740 (2015).

3 Kim, Y. H., Hwang, H. S., Kim, Y. T., Kim, H. S. & Park, Y. W. Modulation of matrix metalloproteinase secretion by adenosine A3 receptor in preeclamptic villous explants. *Reprod Sci* **15**, 939-949, doi:10.1177/1933719108322431 (2008).

4 Song, G. *et al.* The metabolic role of the CD73/adenosine signaling pathway in HTR-8/SVneo cells: A Double-Edged Sword? *Heliyon* **10**, e25252, doi:https://doi.org/10.1016/j.heliyon.2024.e25252 (2024).

5 Jadhav, A. A. & Jain, A. Adenosine deaminase activity in normal pregnancy and pregnancy associated disorders. *Arch Physiol Biochem* **119**, 88-91, doi:10.3109/13813455.2013.777078 (2013).

6 Gaiday, A., Tussupkaliyev, A., Bermagambetova, S., Sakhanova, S. & Dinets, A. Prognostic role of increased serum homocysteine concentration in preeclampsia. *Hypertens Pregnancy* **40**, 118-123, doi:10.1080/10641955.2021.1884257 (2021).

7 Khot, V. V., Chavan-Gautam, P., Mehendale, S. & Joshi, S. R. Variable Methylation Potential in Preterm Placenta: Implication for Epigenetic Programming of the Offspring. *Reproductive Sciences* **24**, 891-901, doi:10.1177/1933719116671001 (2017).

8 Ehlers, L. *et al.* Surface AMP deaminase 2 as a novel regulator modifying extracellular adenine nucleotide metabolism. *The FASEB Journal* **35**, e21684, doi:https://doi.org/10.1096/fj.202002658RR (2021).

9 Liu, B. *et al.* Phosphoribosyl Pyrophosphate Amidotransferase Promotes the Progression of Thyroid Cancer via Regulating Pyruvate Kinase M2. *Onco Targets Ther* **13**, 7629-7639, doi:10.2147/ott.S253137 (2020).

10 Saidak, Z. *et al.* A pan-cancer study of the transcriptional regulation of uricogenesis in human tumours: pathological and pharmacological correlates. *Bioscience Reports* **38**, doi:10.1042/bsr20171716 (2018).

11 Townsend, M. H. *et al.* Overexpression and surface localization of HPRT in prostate cancer provides a potential target for cancer specific antibody mediated cellular cytotoxicity. *Experimental Cell Research* **403**, 112567, doi:https://doi.org/10.1016/j.yexcr.2021.112567 (2021).

12 Shakartalla, S. B. *et al.* 1H-NMR metabolomics analysis identifies hypoxanthine as a novel metastasis-associated metabolite in breast cancer. *Scientific Reports* **14**, 253, doi:10.1038/s41598-023-50866-y (2024).

13 Liu, N. Q. *et al.* Prognostic significance of nuclear expression of UMP-CMP kinase in triple negative breast cancer patients. *Sci Rep* **6**, 32027, doi:10.1038/srep32027 (2016).

14 Luo, J. *et al.* Comprehensive Pan-cancer Analysis of CMPK2 as Biomarker and Prognostic Indicator for Immunotherapy. *Curr Cancer Drug Targets*, doi:10.2174/0115680096281451240306062101 (2024).

15 Li, G., Li, D., Wang, T. & He, S. Pyrimidine Biosynthetic Enzyme CAD: Its Function, Regulation, and Diagnostic Potential. *International Journal of Molecular Sciences* **22**, 10253 (2021).

16 Pfeiffer, C. *et al.* CTPS1 is a novel therapeutic target in multiple myeloma which synergizes with inhibition of CHEK1, ATR or WEE1. *Leukemia* **38**, 181-192, doi:10.1038/s41375-023-02071-z (2024).

17 Hu, L. *et al.* Dietary nucleotides supplementation during the suckling period improves the antioxidative ability of neonates with intrauterine growth retardation when using a pig model. *RSC Adv* **8**, 16152-16160, doi:10.1039/c8ra00701b (2018).

18 Boukalova, S. *et al.* Dihydroorotate dehydrogenase in oxidative phosphorylation and cancer. *Biochimica et Biophysica Acta (BBA) - Molecular Basis of Disease* **1866**, 165759, doi:https://doi.org/10.1016/j.bbadis.2020.165759 (2020).

19 Hamamoto, Y. *et al.* Orotate phosphoribosyltransferase is overexpressed in malignant pleural mesothelioma: Dramatically responds one case in high OPRT expression. *Rare Dis* **4**, e1165909, doi:10.1080/21675511.2016.1165909 (2016).

20 Mazzu, Y. Z. *et al.* A Novel Mechanism Driving Poor-Prognosis Prostate Cancer: Overexpression of the DNA Repair Gene, Ribonucleotide Reductase Small Subunit M2 (RRM2). *Clinical Cancer Research* **25**, 4480-4492, doi:10.1158/1078-0432.Ccr-18-4046 (2019).

21 Li, J., Ye, J., Zhu, S. & Cui, H. Down-Regulation of Phosphoribosyl Pyrophosphate Synthetase 1 Inhibits Neuroblastoma Cell Proliferation. *Cells* **8**, doi:10.3390/cells8090955 (2019).
